# *Cas9*-expressing cattle using the *PiggyBac* transposon all-in-one system

**DOI:** 10.1186/s12864-025-11381-8

**Published:** 2025-03-05

**Authors:** Dong-Hyeok Kwon, Gyeong-Min Gim, Soo-Young Yum, Kyeong-Hyeon Eom, Song-Jeon Lee, Sang-Eun Han, Hee-Soo Kim, Hyeong-Jong Kim, Woo-Sung Lee, Woo-Jae Choi, Ji-Hyun Lee, Do-Yoon Kim, Dae-Jin Jung, Dae-Hyun Kim, Jun-Koo Yi, Byeong-Ho Moon, Won-You Lee, Goo Jang

**Affiliations:** 1 Department of Theriogenology, College of Veterinary Medicine and the Research Institute for Veterinary Science, Seoul National University, Seoul, 08826 Republic of Korea; 2 Bk21 FOUR Future Veterinary Medicine Leading Education & Research Center, Seoul National University, Seoul, 08826 Republic of Korea; 3 LARTBio Inc, Seoul, 14322 Republic of Korea; 4 Embryo Research Center, Seoul Milk Coop, Gyeonggi-Do, 12528 Republic of Korea; 5 Gyeongsangbukdo Livestock Research Institute, GyeongSang Buk-Do, Yeongju, 36052 Republic of Korea; 6 Department of Animal Science, Chonnam National University, Gwangju, 61186 Republic of Korea; 7 School of Animal Life Convergence Science, Hankyong National University, Anseong, Gyeonggi-Do 17579 Republic of Korea

**Keywords:** Bovine embryos, CRISPR-*Cas9*, Genome editing, Knockout, Transgenic cattle

## Abstract

**Background:**

Livestock, particularly cattle, are crucial for biotechnology fields, such as genetic breeding, infectious diseases, bioreactors, and specific disease models. However, genetic engineering in cattle has lagged due to long gestation periods, single embryo pregnancies, and high rearing costs. Additionally, the slow validation of germline transmission and the absence of germline-competent embryonic stem cells hinder progress. With the development of genome editing technologies like *ZFN*, *TALEN*, and *CRISPR-Cas9*, recent advancements have shown that *Cas9*-expressing pigs and chickens have been successfully produced. We hypothesize that generating *CRISPR/Cas9*-expressing cattle and their resources will provide a powerful resource for bovine genome editing, advancing our understanding of bovine genetics and disease resistance.

**Results:**

In this study, two types of *Cas9*-expressing cattle were successfully produced: *Cas9*-*RFP*-fatty acid dehydrogenase I (*FatI*), *Cas9*-*GFP*-sgRNA for the prion protein (sg*PRNP*). Somatic cells from these cattle were induced to mutate multiple target genes when single-guide RNAs (sgRNAs) were transfected into the somatic cells. Additionally, semen from *Cas9* expressing male cattle was frozen and used to fertilize wild-type oocytes, successfully transmitting the transgene (*Cas9*, reporter genes, *FatI*), and sg*PRNP*) to the next generation. Furthermore, the gene editing capabilities of *Cas9*, including knockout and high-efficiency knock-in, were confirmed in embryos derived from F1 semen through in vitro production.

**Conclusion:**

These data demonstrate, for the first time, that *Cas9*-expressing cattle were successfully born, and this transgene was transmitted to the next-generation calves (F1) and F2 embryos. In addition, somatic and germ cells derived from F0 and F1generations were used to evaluate the potential for gene editing (knockout and knock-in) in multiple genes. *PRNP*-mutated F1 cattle are currently being raised as a resistance model for bovine spongiform encephalopathy. These transgenic bovine models and their derivatives will serve as a valuable resource for both in vitro and in vivo genome editing, advancing our genetic understanding of bovine genomics and diseases.

**Supplementary Information:**

The online version contains supplementary material available at 10.1186/s12864-025-11381-8.

## Background

Livestock (sheep, cattle, goats, pigs, etc.) have long been a focus of biotechnology fields, such as genetic breeding [1], infectious diseases [2], bioreactors [3], and specific disease models [4]. Among these livestock, cattle are an important resource for humans, providing essential products such as milk and meat [5]. Owing to these factors, they have been of particular interest to researchers studying genomics, disease resistance [6], milk composition [7], and the secretion of specific proteins using genome engineering [3, 5]. Despite their importance, the genetic engineering of cattle has progressed slowly to date because cattle have a long gestation period (285 days vs. 20 days in mice), typically produce one embryo per pregnancy, and are costly to raise [7, 8]. In addition, there are limited reporter models (i.e., green fluorescent protein [*GFP*] sperm or oocytes) for cattle, validating germline transmission is slow (around 12–18 months), and they have no germline competent embryonic stem cells for successful chimerism in offspring.

Thus, while the advancement and applications of bovine genome editing using various biotechnological approaches are known to be important, its progress has been relatively slow compared to other animals. *Cas9*-expressing pigs and chickens have recently been produced, and these models have been excellent resources for various genome editing applications in chickens and pigs [8]. Moreover, in our previous studies, transgenic cattle with long-term health and germline transmission via transposon-mediated gene transfer proved that transgenic somatic or germ cells can be applied to reproduce genome-edited cattle [9–12]. Additionally, studies have shown that myostatin (*MSTN*)-edited cattle can be born [13], and their mutation is transmitted to the next generation [14]. To expand this work, a bovine genome editing model is needed.

Accordingly, in this study, we hypothesized that cattle expressing CRISPR/Cas9, along with a reporter fluorescence gene, FatI (a gene involved in the regulation of omega-3 and omega-6 fatty acid synthesis), or single-guide RNA (sgRNA) targeting PRNP (a gene associated with prion diseases such as bovine spongiform encephalopathy), could be successfully produced. Furthermore, we proposed that confirming germline transmission in these cattle would validate their genetic stability. These transgenic cattle would then serve as a valuable resource for various applications in the field of bovine genome editing.

## Materials and methods

### Preparation of all-in-one CRISPR-Cas9 plasmid DNAs

Streptococcus pyogenes Cas9 (Sp*Cas9*) complementary DNA (cDNA) (kindly donated by Toolgen [South Korea]), red fluorescent protein (*RFP*) or green fluorescent-gene (*GFP*) was cloned by PCR to construct two all-in-one CRISPR-Cas9 plasmid vectors. One is for *Cas9* expression combined with *FatI* expression. *FatI* DNAs based on NCBI database (Sequence ID: MK208995.1) was synthesized and it was cloned with EF1α promoter. *Cas9*-Puro-*RFP*, EF1α, and *FatI* were cloned into the same *PiggyBac* (PB) expression vectors (PB- *Cas9*-*RFP*-*FatI*). The other is for all-in-one for *PRNP* knock-out. *Cas9* with two constitutive promoters (CAGs and EF1α) and sgRNA for *PRNP* (U6 promoter) was cloned into PB transposon system [10], PB-*Cas9*-*GFP*-sg*PRNP*. The PB systems (pCy43 and PB-CA) were purchased from Addgene (http://www.addgene.org, Plasmid #20,960, USA).

### In vitro* maturation (IVM), *in vitro* fertilization (IVF), and *in vitro* culture of embryos*

This study was conducted following the methodology described by Yum et al. [10]. Ovaries were obtained from a local abattoir, kept in saline at 35 °C, and transported to the laboratory. Cumulus–oocyte complexes (COCs) from follicles 2 to 8 mm diameter were aspirated. For IVM, the selected COCs were cultured in TCM-199 based medium for 22 h at 38.5 °C in a 5% CO₂.

A method of motile spermatozoa separation and purification carried out using the Percoll gradient method [15]. Then, the fertilized oocytes were denuded and cultured in the two-step defined culture medium at 38.5 °C in an atmosphere of 5% O₂, 5% CO₂, and 90% N2 [13, 14]. Briefly, the active motile spermatozoa from the pellet were added to the droplets with matured oocytes. Oocytes were inseminated on day 0 with 1–2 × 10^6^ spermatozoa/ml for 18 h in IVF-TALP medium (Nutricell) under mineral oil. Then, the fertilized oocytes were denuded and cultured in the two-step defined culture medium at 38.5 °C in an atmosphere of 5% O₂, 5% CO₂, and 90% N2 [16, 17].

### Transposon vector microinjection into bovine zygotes

Immature oocyte from ovaries in local slaughterhouse was matured for around 22–24 h and fertilized with frozen-thawed semen. On 15 h after fertilization, presumptive zygotes were selected, and the all-in-one vector (PB- *Cas9*-*RFP*-*FatI* or PB-*Cas9*-*GFP*-U6-sg*PRNP*) and vector form PB transposase (provided by the Sanger Institute [Hinxton, UK]) were microinjected targeting cytoplasm by the injector machine (Femtojet®, Eppendorf, Germany) with 350 hPa of injection pressure (Pi) and 35 hPa of constant pressure (Pc) condition.

### Embryo culture and embryo transfer

After microinjecting the DNAs to Zygotes, surviving embryos were cultured in two step chemically defined culture medium at 38 °C humidified 5% CO₂, 5% O₂ conditions [16]. On day 7, developing embryos were exposed under the fluorescence microscope (Nikon, Japan). A reporter gene (*GFP* or *RFP*) expressing compact morula or blastocyst stage embryo was transferred into synchronized recipients.

### Animal source and experimental procedures

All animals used in this study were produced and reared at the Gyeongsangbuk-do Livestock Research Institute under the approval of the Institutional Animal Care and Use Committee (IACUC #106). The entire process, including embryo transfer for animal production, rearing, genotyping via tissue sampling, blood collection, and sperm collection, was conducted within the institute in accordance with institutional guidelines. Trained personnel performed all procedures under veterinary supervision to ensure animal welfare and ethical integrity. For euthanasia, a humane and ethically approved protocol was followed under veterinary supervision. The animals were first sedated with xylazine (0.15 mg/kg, intravenous injection, Bayer, Germany) to induce deep sedation and muscle relaxation. Once adequate sedation was confirmed, T-61 (5 mL/50 kg, slow intravenous administration, MSD animal health, Rahway, NJ, USA) was administered via the jugular vein until respiratory and cardiac arrest occurred. The absence of vital signs was verified by confirming the cessation of heartbeat, respiration, and corneal reflex. This procedure was conducted in strict accordance with institutional and national ethical guidelines.

### Primary cell culture

Tissue samples were collected from all experimental animals, including 11 F0 (4 PB-*Cas9*-*RFP*-*FatI* and 7 PB-*Cas9*-*GFP*-*sgPRNP*) and 8 F1 offspring (4 PB-*Cas9*-*RFP*-*FatI* and 4 PB-*Cas9*-*GFP*-*sgPRNP*). The ear skin tissue, measuring 0.5 cm in diameter was isolated using biopsy punch. The tissue washed more than three times using phosphate-buffered saline (PBS; Gibco, Cat No. 10010023), containing 1% penicillin/streptomycin (P/S; Gibco, Cat No. 15070063), and was chopped with surgical blade (#10) as small as possible. The copped tissues were incubated at 37 °C for 16 h in the Hank's Balanced Salt Solution (HBSS) with collagenase IV (Gibco, Cat No. 17104019). One week later, after observing outgrowing skin fibroblasts without contamination, the culture dish was re-filled with fresh culture medium (Dulbecco's Modified Eagle Medium [DMEM] supplemented 10% fetal bovine serum [FBS], 1% Non-Essential Amino Acids [NEAA], 100 mM Beta-mercaptoethanol and 1% P/S). As the cells became confluent, the cells were sub-cultured and frozen.

### PCR and end-point RT-PCR

To verify the successful integration of *Cas9* and *FatI*, genomic DNA from the cultured cells was extracted using genomic DNA extract kit (Qiagen, Cat No. 69506). Using specific PCR primer on *Cas9* and *FatI*, PCR amplification was carried out. The PCR products was loaded into 1% agarose gel with DNA ladder. To confirm the expression of the inserted *Cas9* sequence at the level of RNA transcription, total RNA was extracted using a RNA extraction kit (Qiagen, Cat No. 74126), followed by cDNA synthesis (TAKRA, Cat No. 639543). PCR was performed using primers specifically designed for *Cas9* (F: GTTCCATTGACGAGCCAGAT and R: CTGCTCAAAAATGCTGTCCA), with the synthesized cDNA serving as the template. In addition, GAPDH (F: CCACCCAGAAGACTGTGGAT and R: TTGAGCTCAGGGATGACCTT) was used as a housekeeping gene.

### SDS PAGE and western blotting

Isolated primary cells were lysed by 200 µl of RIPA buffer (Tris–HCl, pH 7.5; 50 mM; SDS, 0.1%; Triton X-100, 1%; NaCl, 150 mM; Sodium deoxycholate, 0.5%; and EDTA, 2 mM; Cat No. BR002, BIOSOLUTION, Republic of Korea) with Pi cocktail (cOmpleteTM, Mini, EDTA-free Protease Inhibitor Cocktail, Cat No. 11836170001, Roche, Switzerland), followed by centrifugation at 13,000 rpm for 10 min. Then, the supernatant was used for total protein extract quantification. Bradford solution (Quick StartTM Bradford Protein Assay Kit 1, Cat No. 5000201, Bio-Rad, US) and *BSA* (Bovine serum albumin solution, Quick StartTM Bradford Protein Assay Kit 1, Cat No. 5000201, Bio-Rad, US) were used for protein quantification. And then, quantified proteins were mixed with protein sample buffer (Bromophenol blue 0.1%, Dithiothreitol 0.5 M, Glycerol 50%, SDS 10%, Tris–HCl pH 6.8, 150 mM). The protein samples were loaded on the 10% (w/v) acrylamide gel (Mini-PROTEA TGX Gels, Cat No. 467033, Bio-Rad, US) and transferred onto a polyvinylidene fluoride membrane by the wet/tank transfer BioRad system (Mini Trans-Blot Electrophoretic Transfer Cell, Cat No. 1703930, Bio-Rad, US). The membrane containing protein samples was incubated with a blocking solution containing TBS-T buffer and 5% (w/v) skim milk for 1 h. Next, the membrane was incubated with anti-*Cas9* mouse antibody (1:1,000 dilution; 7A9-3A3, Cat No. ab191468, abcam, UK) or anti-β-actin mouse antibody (1:1,000 dilution; C4, Cat No. sc-47778, Santa Cruz, US) at 4 °C overnight on the 50 rpm shaker. After this step, membrane containing protein samples and primary antibody was washed with TBS-T buffer three times for 5 min each wash. After washing, the membrane was incubated with anti-mouse goat HRP conjugated antibody (1:5,000 dilution; Cat No. A90-116P, BETHYL, US) at room temperature for 1 h on the 50 rpm shaker. Then, the membrane was washed with TBS-T buffer three times for 5 min each wash. Finally, the membrane was incubated with ECL reagents (WesternBright®, advansta, US), and then the chemiluminescence image was captured by using iBright1500 (Thermo Fisher Scientific, US).

For stripping the protein on the membrane, the detected membrane was incubated with stripping buffer (Glycine 1.5% [w/v], SDS 0.1% [w/v], Tween-20 1% [v/v], pH 2.2) for 5 ~ 10 min in two times. Then, the membrane was washed with TBS-T buffer three times for 5 min each wash. It is ready to block.

### Transfection of sgRNA and mutation assay

In somatic cells from the transgenic cattle, to know whether *Cas9* is active or not, only sgRNAs for various target genes were designed using Cas9-Designer (http://www.rgenome.net/cas-designer). sgRNA plasmids were transfected into the fibroblasts using Neon® Transfection system (Invitrogen Cat No. MPK5000). For each transfection, 3 × 10^5^ cells were used, and the experiment was performed under the conditions of optimization No. 16 (Voltage: 1400 V, width: 20 ms, pulses: 2 pulses). On 48 h later, the growing cells were harvested and used for genomic DNA extracts. Using the genomic DNAs, target specific PCR was carried out and the products were reacted with T7E1 enzyme for knowing mutation existence on target region. The sequences of the primers used in this study are provided in (Supplemental Table S2).

### Somatic cell nuclear transfer (SCNT) and Microinjection of gRNA

For the preparation of donor cells, fibroblasts were extracted from the ear tissues of the *Cas9*-*FatI* expressing cattle using the primary cell culture technique mentioned previously. These cells were then preserved in a freezing medium and stored in liquid nitrogen at −196 °C until they were utilized for SCNT. The donor cells, at passages 4 to 6, were employed for SCNT. Before the SCNT process, the cells were thawed and cultured for 3 to 4 days until they reached 100% confluence to ensure contact inhibition. They were then separated from the monolayer by trypsinizing for 30 s. Following the preparation of donor cells for SCNT, oocytes were matured for 22 h. The cumulus cells were then denuded by repeated pipetting in 0.1% hyaluronidase in HEPES-buffered TCM-199. Subsequently, the metaphase and first polar body were removed. The transfer of a donor cell, derived from the ear tissue of the *Cas9*-*FatI* expressing cattle, into an enucleated oocyte was performed according to the method previously described [18]. The reconstructed embryos were fused and activated for 4 min using ionomycin, followed by a 4-h incubation in 1.9 mM 6-dimethylaminopurine. For interferon-tau (*IFNT)* knockout, the fused oocytes were microinjected with sgRNA for *IFNT* in its messenger RNA (mRNA) form. Cloned embryos were incubated in 25 μL microdrops of chemically defined media, covered with mineral oil, for 8 days at 38.5 °C in an environment containing 5% O₂, 5% CO₂, and 90% N₂. Cleaved embryos and blastocysts were observed on days 4 and 8 of culture, respectively. The number of cells in SCNT blastocysts was determined using Hoechst 33342 staining.

### Adeno-Associated Virus 6 (AAV6) embryo infection

Through the use of transgenic cattle-derived semen expressing *Cas9*, gene editing was performed by IVF using this semen and by treating AAV6, which contains gRNAs or a knock-in donor sequence, into the media to facilitate infection and subsequent gene editing. AAV6 was treated to D2 media at a concentration of 5 × 10^9^ GC/µl, spanning from the eight-cell stage to the blastocyst stage (72 h). For knockout applications, a AAV6 containing sgRNA for *PRNP* sequences (under the U6 promoter) was employed. This AAV6 was specifically designed and produced by company (Vigene Biosciences, US). For the knock-in process, two AAV6s were co-infected into the media: one carrying the gRNA for the BSA sequence, and another containing the AfIII enzyme site and Attb within 500 bp ARMs of the BSA target sequence. Both AAV6s were specifically designed and produced by company (GenCopoeia, US).

### Freezing semen

To validate the germline transmission of *Cas9*, reporter gene (*GFP* or *RFP*), and *FatI*, semen collected from male transgenic cattle at 18 months old using an artificial vaginal (Fujihira Industry, Tokyo, Japan) containing warm water at 50–55 °C as previous our study [9]. After collecting the semen, it was transported immediately into the laboratory for freezing. The semen was diluted 50%:50% using OPTIXcell (IVM technologies, France) and kept at room temperature for 10 min. Thereafter, the first diluted semen was diluted again 50%:50% and a sperm concentration of 5.0 × 10^7^/ml was kept at 4 °C for 2 h. The concentrated sperm was loaded into a 500 µl semen straw (IMV technologies, France) and sealed with straw powder (Fujihira Industry, Tokyo, Japan). The straw was frozen above 5.0 cm from liquid nitrogen surface for 30 min and then plunged into a liquid nitrogen tank.

## Results

### Production of transgenic cattle (F0)

**Fig. 1:**
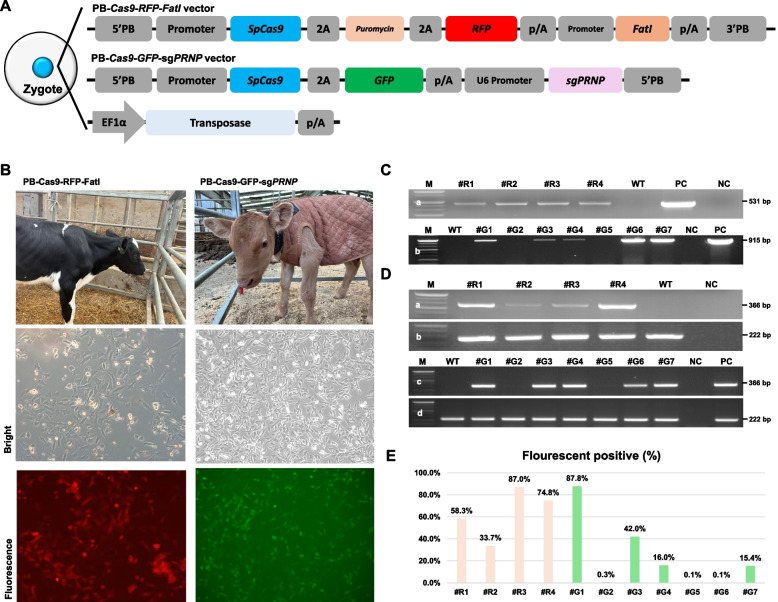
Generation and analysis of transgenic cattle via *PiggyBac* (PB)-mediated all-in-one vectors. **A** Schematic representation of the microinjection process for generating transgenic cattle. Two PB-mediated all-in-one vectors, PB-*Cas9*-*RFP*-*FatI* and PB-*Cas9*-*GFP*-sg*PRNP*, were microinjected into in vitro fertilized embryos along with a transposase vector to facilitate genomic integration. **B** Production of gene-edited calves and fluorescence observation in ear tissue-derived fibroblasts. **C** Integration of transgene in transgenic cattle. Genomic analysis showed successful transgene integration in all PB-*Cas9*-*RFP*-*FatI* cattle (a), while in PB-*Cas9*-*GFP*-sg*PRNP* cattle, integration was confirmed in calves #G1, #G3, #G4, #G6, and #G7 (b). **D** *Cas9* mRNA expression levels in transgenic calves. end-point PCR results indicated *Cas9* mRNA expression only in calves where transgene integration was confirmed (a: end-point PCR of Cas9 in PB-Cas9-RFP-FatI cattle, b: end-point PCR of GAPDH in PB-Cas9-RFP-FatI cattle, c: end-point PCR of Cas9 in PB-Cas9-GFP-sgPRNP cattle, d: end-point PCR of GAPDH in PB-Cas9-GFP-sgPRNP cattle). **E** Quantification of fluorescence-positive cells among transgenic cattle cells. The ratio of fluorescence-positive cells, indicative of successful gene editing and expression, was measured, demonstrating the effectiveness of the CRISPR/*Cas9* system in creating transgenic models. (M, marker; WT, wild type; NC, negative control; PC, positive control)

Two all-in-one PB vectors (PB-*Cas9*-*RFP*-*FatI* and PB-*Cas9*-*GFP*-sg*PRNP*) were microinjected into in vitro fertilized embryos. To produce F0 cattle expressing *Cas9*, red fluorescent protein (*RFP*), and fatty acid dehydrogenase I (*FatI*), PB-*Cas9*-*RFP*-*FatI* was microinjected with a transposase vector into in vitro fertilized embryos (Fig. 1A). A total of 794 oocytes were microinjected and 151 blastocysts developed. Of the 151 blastocysts, 34 expressed *RFP*. Blastocysts expressing *RFP* were selected and transferred into five recipients (with a single blastocyst per recipient). Four calves (#R1, #R2, #R3, and #R4) were born without any assistance from a husbandry technician or veterinarian (Fig. 1B, Table 1).

**Table 1:** Overview of microinjections and fluorescence-positive embryo transfer for *Cas9*-expressing cattle production

Group	No. injected oocyte	No. Blastocyst	No. *RFP* or *GFP* Blastocyst	No. recipient	No. offspring
PB-*Cas9*-*RFP*-FatI	794	151	34	5	4
PB-*Cas9*-*GFP*-sg*PRNP*	424	84	31	18	7

From the microinjection of 794 oocytes with the PB-*Cas9*-*RFP*-*FatI* vector, 151 blastocysts were developed and 34 exhibited *RFP* expression. A single *RFP*-positive blastocyst was transferred into each of five recipient animals, resulting in the birth of four calves. In the other group, the PB-*Cas9*-*GFP*-sgPRNP vector was microinjected alongside a transposase vector into 424 zygotes, yielding 84 blastocysts, 31 of which expressed *GFP*. One *GFP*-expressing blastocyst was transferred into each of 18 recipients, leading to seven confirmed pregnancies. Five pregnancies resulted in normal births, while two (#G1 and #G6) resulted in stillbirths due to dystocia

Another all-in-one vector (PB-*Cas9*-*GFP*-sg*PRNP*) was microinjected with transposase vector into 424 zygotes, and 84 blastocysts developed. Of the 84 blastocysts, 31 expressed *GFP* and were transferred into 18 recipients. Seven fetuses were confirmed; five were born normally (#G2, #G3, #G4, #G5, and #G7), while two (#G1 and #G6) were stillborn due to dystocia (Fig. 1B, Table 1).

### Analysis of the genomic integration and the expression ratio of the transgenes

Genomic PCR was used to detect *Cas9* in all of the transgenic calves, and the transgene expression ratio was evaluated using end-point RT-PCR and fluorescence-activated cell sorting (FACS). Transgene integration was observed in all PB-*Cas9*-*RFP*-*FatI* cattle, whereas in PB-*Cas9*-*GFP*-sg*PRNP* cattle, it was only observed in #G1, #G3, #G4, #G6, and #G7 (Fig. 1C). *Cas9* mRNA expression, confirmed by end-point PCR, was observed only in the transgenic calves with transgene integration (Fig. 1D). The ratio of fluorescence-positive cells was measured, with the highest and lowest values observed in the #R group (87.0% and 33.7%, respectively) and the #G group (87.8% and 0.1%, respectively). Detailed data for all samples are presented in Fig. 1E.

The *PRNP* mutation ratio in transgenic cattle with PB-*Cas9*-*GFP*-sg*PRNP* was analyzed by deep sequencing. Calves #G1–#G7 showed mutations on *PRNP* at 4.1%, 0.0%, 48.3%, 0.2%, 0.0%, 99.6%, and 94.4%, respectively. These results indicate diverse mutation patterns in the targeted *PRNP* locus, as shown in Table 2. By comparing the *GFP* FACS results with the deep sequencing data, we found that the *GFP* expression rate differs from the *PRNP* mutation rate. To determine the off-target effect on the genome, the candidate sites were analyzed for the presence of mutations using a T7E1 assay, and the results showed that there were no off-target effects (Supplemental Figure S1).

**Table 2:**
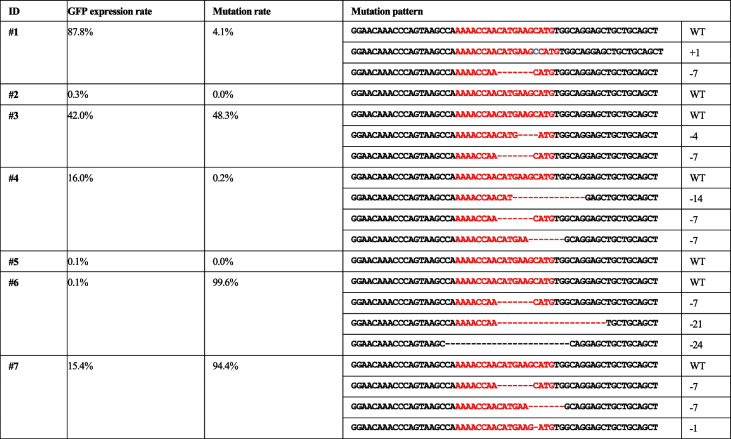
PRNP mutation ratios in PB-Cas9-sgPRNP cattle using deep sequencing

Calves #G1–#G7 exhibited PRNP mutation ratios of 4.1%, 0.0%, 48.3%, 0.2%, 0.0%, 99.6%, and 94.4%, respectively. These values indicate the proportion of cells in each calf where the PRNP gene was successfully edited (red letter: target sequence for PRNP)

## Germ line transmission

### F0-Cas9-RFP-FatI

**Fig. 2:**
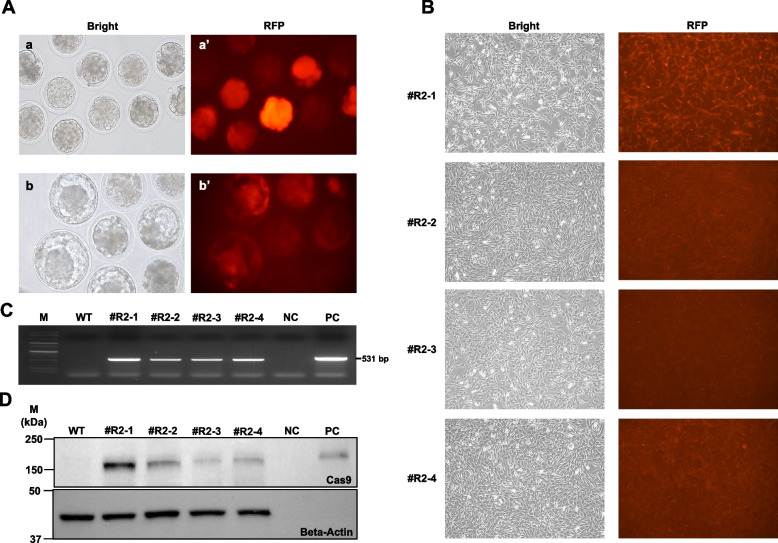
Developmental and genetic analysis of F1 PB-*Cas9*-*RFP*-*FatI* cattle expressing *RFP* and *Cas9* proteins. **A** Representative images showing Development Day 4 (a and a') and Day 7 (b and b') embryos fertilized using frozen/thawed sperm from a F0 transgenic male bull (#R2). *RFP* expression was monitored, and the successful gene expression was confirmed early in development. **B ***RFP* expression in primary cells from F1 PB-*Cas9*-*RFP*-*FatI* cattle. Image demonstrating *RFP* expression in primary cells derived from all transgenic calves, which confirms the persistence of transgene expression. **C** *Cas9* sequence locus amplified by PCR, showing transgene integration in the genome of the calves, verifying that genetic modification was successfully inherited. **D** Western blot results showing the presence of *Cas9* protein in samples from all calves, which confirms ongoing expression of *Cas9* for intended gene editing functions

Since one male transgenic cow (#R2) reached puberty, semen was collected and frozen for long-term storage, and germline transmission was confirmed through IVF with wild-type oocytes. The frozen/thawed sperm from the male transgenic cow was fertilized with in vitro matured oocytes from a wild-type cow. *RFP* expression was observed in morulae 4 d after culture and in blastocysts 7 d after culture (Fig. 2A). Selected preimplantation embryos with *RFP* expression were transferred to four surrogate mothers, and three male calves (#R2-1, #R2-2, #R2-3) were born. One male (#R2-1) suffered from chronic tympany and was euthanized at age 11 months. The other two male calves (#R2-2, #R2-3) have been growing without any health issues.

Additionally, #R4 (female) became pregnant by #R2 (male) via natural breeding. At the end of the pregnancy, a veterinarian decided to induce parturition. After treating induced parturition, a male calf (#R2-4) was successfully born. The calf was euthanized due to an inability to stand, a condition caused by cerebellar hypoplasia that was diagnosed as a result of bovine viral diarrhea virus infection.

Primary cells from all transgenic calves showed *RFP* expression (Fig. 2B), transgene *Cas9* mRNA expression and *FatI* sequence integration were observed (Fig. 2C and Supplemental Figure S2A), and *FatI* mRNA expression was also detected (Supplemental Figure S2B). Additionally, the *Cas9* protein was detected in all calves in the Western blot results (Fig. 2D). Semen from #R2-2 was collected, and it showed normal fertility along with *RFP* expression in blastocysts during in vitro culture (Supplemental Figure S3).

### F0-Cas9-GFP-sgPRNP

Two calves (#G3 [female] and #G7 [male]) with high *PRNP* mutations have been growing well for over 3 years. To confirm the germline transmission in #G3, the oocytes were collected by ovum pick up (OPU), matured in vitro, and fertilized with wild-type frozen/thawed semen. fter performing OPU, the cells derived from the follicular fluid were cultured and assessed for *PRNP* mutations using the T7E1 assay. Both *PRNP* mutations and *GFP* expression were observed (Supplemental Figure S4A). Through nine OPU sessions, a total of 49 oocytes were collected, of which 14 developed into blastocysts. These blastocysts exhibited both *GFP* expression and *PRNP* mutations (Supplemental Figure S4B). Nine embryos were transplanted into nine recipients, respectively. Ultimately, three cattle became pregnant and gave birth (#G3-1, #G3-2 and #G3-3).

When #G7 (male) reached puberty, its semen was collected and frozen. The frozen/thawed semen was later subjected to a genomic mutation assay. The target locus of the genomic DNA from the semen was positive for *PRNP* mutation (Supplemental Figure S4C). Then, the oocytes from the wild-type cow were fertilized with frozen/thawed semen from F0 #G7 male and cultured for 7 d. All blastocysts were positive for the *PRNP* gene mutation (Supplemental Figure S4D); some blastocysts were selected and transferred into five recipients, and one pregnancy was observed and the animal successfully gave birth to a single calf (#G7-1).

**Fig. 3:**
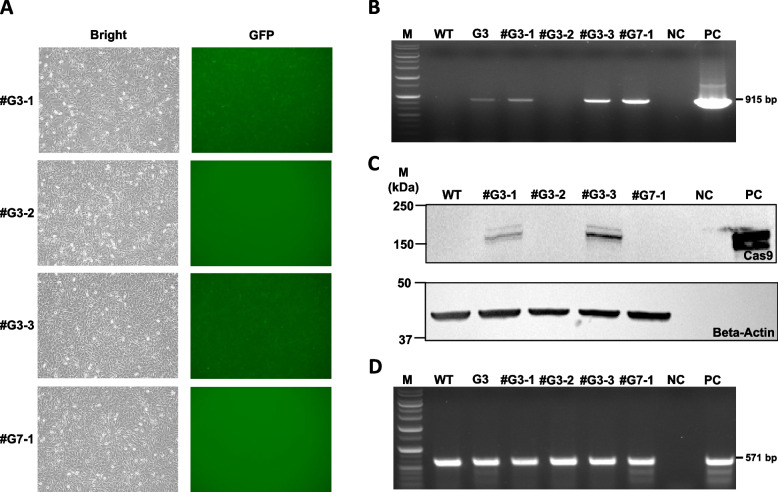
Genetic analyses of F1 PB-*Cas9*-*GFP*-sg*PRNP* calves. **A** *GFP* expression was confirmed in primary cells from the ear tissue of calves #G3-1 and #G3-3. **B** PCR revealed transgenic integration in calves #G3-1, #G3-3, and #G7-1. **C** Western blotting to detect the *Cas9* protein in calves #G3-1 and #G3-3. **D** T7E1 assay revealing that the *PRNP* mutation was exclusively identified in calf #G7-1

All F1 calves (three male calves [#G3-1, #G3-3, and #G7-1] and one female [#G3-2]) were born normally. The male calves (#G3-1 and #G3-3) and the female calf (#G3-2) were derived from #G3 oocytes fertilized with wild-type (WT) semen. The male calf #G7-1 was produced from WT oocytes fertilized with frozen/thawed semen from the F0 male (#G7). Moreover, *GFP* expression was confirmed in #G3-1 and #G3-3 in primary cells derived from ear tissue (Fig. 3A), and transgene integration was observed in #G3-1, #G3-3, and #G7-1 (Fig. 3B). The *Cas9* protein was detected in #G3-1 and #G3-3 using Western blotting (Fig. 3C), whereas only #G7-1 showed the *PRNP* mutation in the T7E1 assay (Fig. 3D).

## Storage and application of the somatic cells and germ cells

### Application of Cas9-expressing somatic cells in gene engineering

**Fig. 4:**
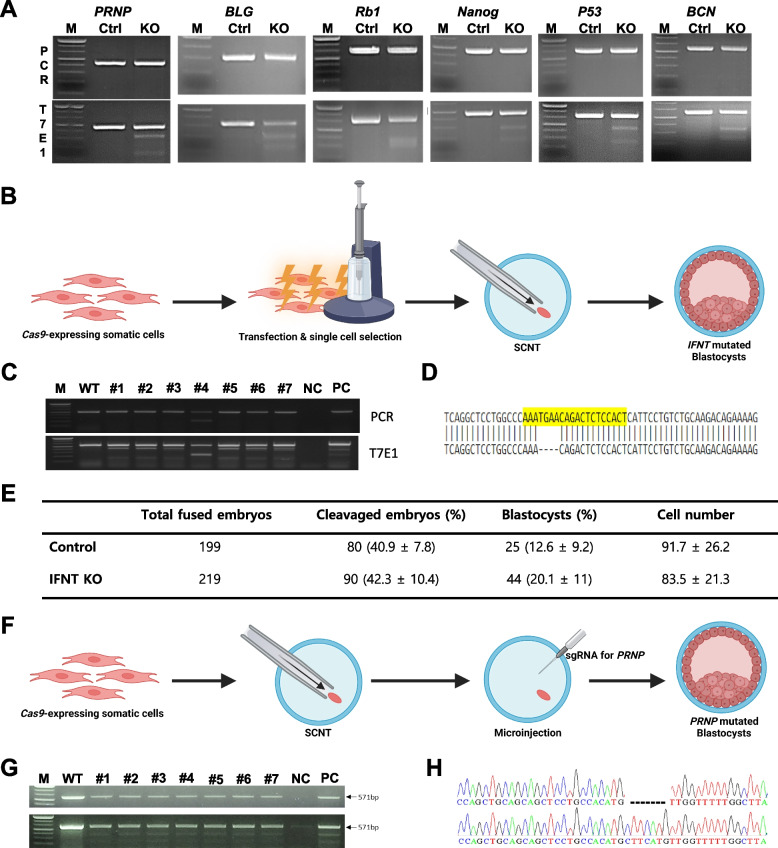
Utilization of *Cas9*-expressing somatic cells for gene knockout and embryonic development via somatic cell nuclear transfer (SCNT). **A** T7E1 assay results showing successful specific gene editing to several genes (*PRNP*, *BLG*, *Rb1*, *NANOG*, *P53*, and *BCN*) using *Cas9*-expressing somatic cells. (M: Marker, Ctrl: #R2-2 somatic cell as control, KO: Ctrl group with gRNA transfection). **B** Schematic design of the procedure for creating *IFNT* mutated blastocysts. *Cas9*-expressing somatic cells are transfected with sgRNA targeting the *IFNT* gene for using as a donor cell of SCNT. **C** *IFNT* mutation detection of Cas9-expressing single cell colonies transfected with *IFNT* sgRNA using T7E1 assay(#1–7 = single cell colony) and (**D**) Sanger sequencing (yellow = sgRNA for *IFNT* target sequence). **E** Table showing pre-embryonic developmental competency of cloned embryos from both groups (Control and *IFNT* KO). **F** Schematic design of the procedure for creating *PRNP* mutated blastocysts. The oocyte-fused with *Cas9*-expressing donor cell is microinjected with sgRNA for *PRNP* for KO blastocyst production. **G** *PRNP* mutation detection of embryo derived from injection of sgRNA targeting *PRNP* after *Cas9*-expressing donor cell fusion using the T7E1 assay. **H** Representative Sanger sequencing result showing a −7 bp deletion in the *PRNP* target region

Since cattle with confirmed *Cas9* expression in the primary somatic cells also had *Cas9* transgene integration, an analysis was conducted to determine whether the presence of specific sgRNAs targeting given genes could induce mutations in those target genes. Mutations were observed in *Cas9*-expressing somatic cells targeting various genes, including *PRNP*, beta-lactoglobulin (*BLG*), retinoblastoma 1 (*Rb1*), Nanog homeobox (*NANOG*), beta-casein (*BCN*), and tumor protein p53 (*P53*), as confirmed by the T7E1 assay (Fig. 4A).

Furthermore, the mutated cells for a specific gene were used as donor cells in SCNT to investigate the gene's functional impact on pre-embryonic development (Fig. 4B). Cas9 cells were transfected with g*IFNT*, resulting in the acquisition of seven viable single-cell colonies, all of which exhibited mutations as confirmed by T7E1 analysis (Fig. 4C). Subsequently, one colony (#1) was selected, and Sanger sequencing confirmed a −4 bp deletion, which was then used as the SCNT donor (Fig. 4D). To evaluate the potential of *Cas9*-expressing somatic cells as donors for SCNT, *Cas9* cells with *IFNT* knockout were created, and their embryonic development was observed. The cloned blastocysts with *IFNT* knockout developed normally to the blastocyst stage with a normal cell number (Fig. 4E), suggesting that once transferred into surrogates cows, a sufficient number of transgenic cattle could be produced for various applications. Additionally, following the fusion with donor cells expressing the *Cas9* gene, cloned blastocysts with *PRNP* mutations were produced via the microinjection of sgRNA for *PRNP* (Fig. 4F). This process resulted in a blastocyst formation rate of approximately 15.5 ± 4.1% and a *PRNP* mutation rate of around 64.2 ± 3.8%. The mutation rate was defined as the proportion of blastocysts carrying at least one mutated allele at the target locus, as determined by T7E1 assay (Fig. 4G and H).

### Application of Cas9-expressing germ cells in gene engineering

Sperm was extracted from male cattle with *Cas9* expression (#G3-1, #G3-3, and #R2-2), and more than 200 straws of frozen semen were stored in a liquid nitrogen tank. The sperm exhibited normal developmental competency and expressed fluorescence (*RFP* and *GFP*) at the blastocyst stage.

**Fig. 5:**
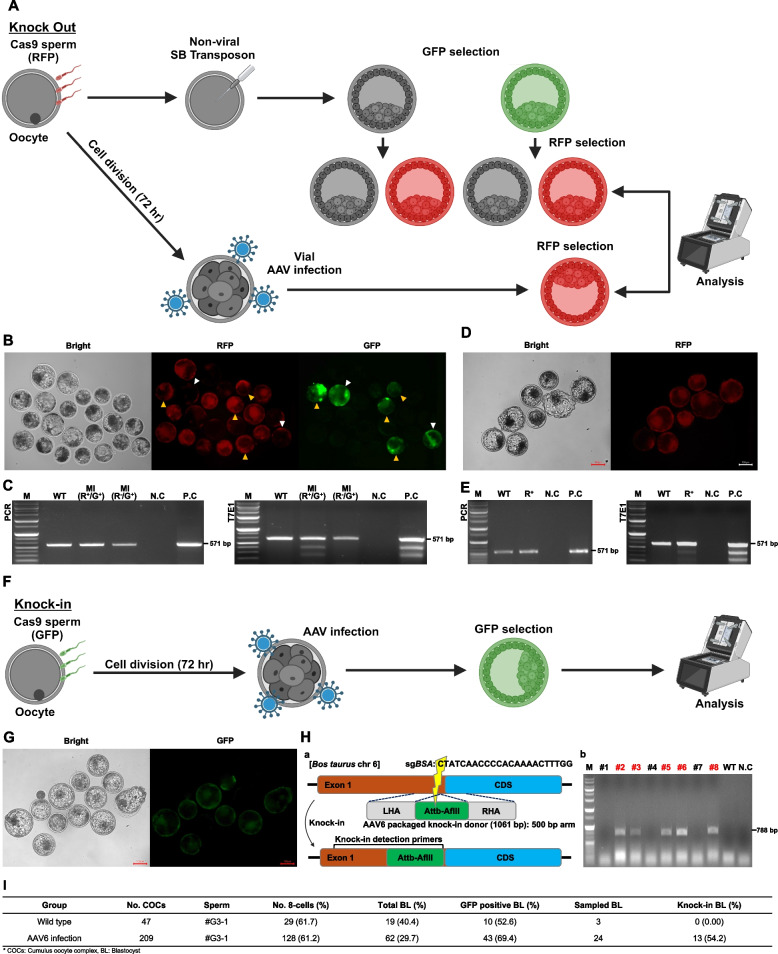
Gene engineering using *Cas9*-expressing germ cells via viral and non-viral methods. **A** Schematic experiment design for testing sgRNA for *PRNP* gene knockout utilizing viral and nonviral approaches without introducing *Cas9* during the pre-embryonic in vitro culture process. **B** An SB transposon vector containing both *GFP* and sg*PRNP* sequences was microinjected, resulting in mutations identified in embryos that expressed both *RFP* and *GFP* (yellow arrow: R + and G + , white arrow: R- and G +). **C** T7E1 assay result confirming *PRNP* mutations (Lanes: M: marker, WT: wild type, MI: microinjection, R + : *RFP* positive, G + : *GFP* positive, R-: *RFP* negative, NC: negative control, and PC: positive control). **D** and **E** The viral method for gene editing was carried out using AAV6 containing an sg*PRNP* sequence. T7E1 assay results of *PRNP* mutation in the *RFP*-positive group cultured for 72 h after media treatment with AAV6. **F** Schematic experiment design for knock-in strategy at the *BSA* gene locus in embryos. **G** Observation of *GFP*-positive blastocysts post-AAV6 infection. **H** Schematic of the knock-in strategy and validation. Diagram showing targeting site on Bos taurus chromosome 6, where sgRNA targets bovine serum albumin (BSA). knock-in donor containing Attb-AfIII sequences for homologous recombination, flanked by 500-bp left and right homology arms (LHA and RHA) (a). PCR validation of the knock-in band. Eight randomly selected *GFP*-positive blastocysts were sampled, and PCR was carried out with primers targeting sequences both inside and outside the knock-in regions (b). **I** Summary table of the knock-in efficiency (results from three experiments) including number of cumulus-oocyte complexes (COCs), 8-cell stage embryos, total blastocysts (BL), *GFP*-positive blastocysts (*GFP* + BL), and percentage of knock-in in sampled blastocysts

The next challenge was to proceed with IVF using *Cas9*-expressing sperm (#G3-1 and #R2-2) to induce knockout or knock-in. To induce knockout using *Cas9* sperm (#R2-2), both viral and nonviral methods were tested (Fig. 5A). For the nonviral method, a *Sleeping Beauty* (SB) transposon vector capable of expressing GFP and sgRNA for *PRNP* was microinjected, and the experiment was conducted in a single attempt using 188 oocytes. A total of 22 blastocysts (11.7%) were developed, among which 14 blastocysts (7.45%) were RFP-positive, and 8 blastocysts (4.26%) were RFP-negative. Among the RFP-positive group, 4 blastocysts (3.39%) were double-positive (RFP and GFP). Separately, 2 blastocysts (1.69%) were GFP single-positive but did not exhibit RFP expression. These blastocysts were categorized based on these fluorescence patterns, and samples were pooled for further analysis. A mutation was observed in the *RFP* and *GFP* double-positive group (Figs. 5B and C). For the viral method, a vector expressing sgRNA for *PRNP* was packaged into an AAV6. The experiment was conducted in a single attempt using 162 oocytes. After 72 h of media treatment of AAV6 from the 8-cell stage to the blastocyst stage, 18 blastocysts (11.1%) were developed. Among these, 8 blastocysts (4.94%) were RFP single-positive. These RFP-positive blastocysts were pooled and sampled for further analysis, and mutations were confirmed in *RFP*-positive blastocysts treated with AAV6 (Figs. 5D and E).

In the case of knock-in, embryos fertilized with #G3-1 sperm were infected with AAV6 carrying sgRNA and a knock-in donor DNA targeting bovine serum albumin (*BSA*) (Fig. 5F). After 72 h of media treatment with AAV6, *GFP*-positive blastocysts were observed and sampled (Fig. 5G). The experiment was conducted with a total of 128 8-cell stage embryos, of which 62 (48.4%) developed into blastocysts. Among these, 43 blastocysts (33.6%) were GFP-positive. In each experiment (repeat time = 3), eight randomly selected *GFP*-positive blastocysts were sampled, resulting in a total of 24 GFP-positive blastocysts analyzed. Among the analyzed blastocysts, 13 were confirmed to have knock-in through PCR using primers targeting sequences inside and outside the knock-in regions, followed by gel electrophoresis. As a result, a knock-in efficiency of 54.2 ± 11.8% was confirmed through Sanger sequencing (Figs. 5H, I and Supplemental Figure S5).

## Discussion

While the application of genome engineering technology in cattle is considered a valuable resources for studying disease resistance, genetic trait improvement, bioreactor production, and basic embryology, progress has been limited due to the absence of germline transmitted embryonic stem cells, the length process required to confirm germline transmission, and the challenges of precise gene editing in both somatic and germ cells [19–22]. In this study, we report that the successful birth of cattle constitutively expressing *Cas9*, a reporter gene (*GFP* or *RFP*), *FatI*, and sgRNA for *PRNP*, a powerful bovine genome editing model, with confirmed germline transmission of the transgene to the next generation.

The main purpose of this study was to identify primary and germ cells from transgenic cattle expressing *Cas9*, as these will be valuable resources for bovine genome editing research. As shown in Fig. 4A, knockout was achieved efficiently at multiple target loci. Those knockout cells can be used in SCNT to study genetic function or knockout offspring either in vitro or in vivo. For example, *IFNT* knockout somatic cells were produced in this study, and SCNT was used to assess their effect on pre-embryonic developmental competence (Fig. 4C). Similarly, various experimental challenges targeting other genes could potentially be conducted.

In *PRNP* knockout cattle, a vector expressing *Cas9* and *GFP* along with sgRNA for *PRNP* was used, and after transferring the *GFP*-expressing blastocysts into recipient cows, *PRNP* mutated cattle were successfully born. However, sgRNA silencing and promoter silencing were observed in the respective individuals (#G2, #G5, #G3-1 and #G3-3). In in vitro experiments, 100% of the cells with *GFP* expression also showed mutations on *PRNP*. However, after embryo transfer and isolation from the resulting offspring, not all *GFP*-positive cells exhibited mutations. It is hypothesized that this phenomenon could be due to interference between the CAG promoter, which expresses *Cas9* and *GFP*, and the U6 promoter, which expresses sgRNA for *PRNP* [23]. To address this issue, the experiment was repeated using the EF1α promoter instead of the CAG promoter. As a result, *GFP* was expressed well at the blastocyst stage, and *PRNP* mutations were confirmed. Although *GFP* expression was not visually apparent in the somatic cells of the born calves, the FACS analysis showed higher *GFP* expression compared to the wild type. In subsequent generations (e.g., F1), *GFP* was not observed in the blastocysts, and 100% heterozygous mutations of *PRNP* was observed (Supplemental Figure S4E). After embryo transfer, a *PRNP* hetero-mutant calf was born. While previous studies on prion knockout cattle have been conducted, successful germline transmission has not yet been achieved [24–26]. The current *PRNP* heterozygous mutated F1 individual (#G7-1), now 24 months old, is growing well without any health issues or abnormalities in blood tests (Supplemental Table S1). In further study, these results will be validated with more F1 offspring, and homozygotic *PRNP* knockout models will be developed to better understand the pathogenesis of bovine spongiform encephalopathy (BSE).

In the application of *Cas9*-*RFP* sperm, when oocytes were fertilized with the sperm, *RFP* expression was observed at the 8–16-cell stage, consistent with our previous studies [9, 27]. As a nonviral method for gene editing, a SB transposon vector carrying *GFP* and sgRNA targeting the desired locus was used to ensure continuous sgRNA expression at 8–16 cell stage. *GFP*-positive blastocysts were selected and analyzed for mutation. As expected, the embryos exhibited the mutation (Fig. 5C), indicating that *Cas9* activity was expressing well at the 8–16-cell stage and beyond. As a viral method for gene editing, after infecting the AAV6 with sgRNA and culturing for 72 h, the formed blastocysts were evaluated, and the mutations were observed. In addition, this study hypothesized that using *Cas9*-expressing sperm could enhance knock-in efficiency and reduce negative effects on embryonic development. The results showed a knock-in efficiency of 54.17% and a blastocyst formation rate of 29.67% in the AAV6 infection group, demonstrating a tendency toward improved outcomes compared to previous studies that reported knock-in efficiency (~ 40%) and blastocyst formation rates (~ 11%) [28] when directly injecting embryos [19, 20]. However, considering potential variations among embryo batches, these results should be interpreted with caution rather than as a definitive trend. Furthermore, the potential impact of AAV6 on mosaicism remains an important consideration for further studies. Comprehensive analyses, including single-cell sequencing and lineage tracing, will be necessary to evaluate the extent and distribution of mosaicism in embryos edited using AAV6.

In our previous study, several transgenic cattle with transposon-mediated gene delivery were born, grew up well, and transmitted the gene to the next generation without any health issues [10, 12, 19]. However, in this study, although the blood analysis revealed no specific abnormalities, the coincidental occurrence of diseases was relatively high. This resulted in the euthanasia and death of a few transgenic cattle even though there was no detectable phenotype in Cas-expressing mice [29–31]. It might be related to an error caused by genome instability or the position of integration. A limited number of samples were subjected to analysis, so we need to analyze more samples to identify the long-term effects of *Cas9* expression (i.e., for more than three generations).

## Conclusion

In conclusion, we demonstrated that cattle expressing *Cas9* with *FatI* or sgRNA for *PRNP* by transposon gene delivery were born and grew up to reach puberty. Moreover, for the first time, the transgene of those cattle was transmitted into the germline of the next generation, resulting in the birth of several F1 cattle, including offspring for the BSE resistance model, and they are currently growing well (age: 24 months) and in good health. The somatic and germ (sperm) cells from these transgenic cattle functionally expressed *Cas9*, which suggests a more straightforward and convenient approach for researchers to perform gene editing, including efficient knock-in. Utilizing these resources (somatic and germ cells) may enable the induction of bovine genome engineering with AAVs expressing one or libraries of sgRNA, proving a valuable tool for both agricultural and veterinary genomic editing.

## Supplementary Information

Supplementary Material 1.

Supplementary Material 2.

## Data Availability

We have submitted the Deep-sequencing (PRJNA1187360) and Sanger sequencing (PRJNA1187378 and PRJNA1208189) data to the NCBI SRA. And, this study includes Western blot analysis of proteins corresponding to the UniProt accession numbers Q99ZW2 and P60712, with the datasets available in the UniProt repository.

## Abbreviations

CRISPR: Clustered Regularly Interspaced Short Palindromic Repeats
*Cas9*: CRISPR-associated protein 9
sgRNA: Single-guide RNA
*PRNP*: Prion Protein
*FatI*: Fatty Acid Dehydrogenase I
PCR: Polymerase Chain Reaction
SDS PAGE: Sodium Dodecyl Sulfate Polyacrylamide Gel Electrophoresis
*RFP*: Red Fluorescent Protein
*GFP*: Green Fluorescent Protein
IVF: In Vitro Fertilization
SCNT: Somatic Cell Nuclear Transfer
AAV6: Adeno-Associated Virus type 6
TCM-199: Tissue Culture Medium-199
FBS: Fetal Bovine Serum
TBS-T: Tris-buffered Saline with Tween 20
*BSA*: Bovine Serum Albumin
DMEM: Dulbecco's Modified Eagle Medium
PBS: Phosphate-buffered Saline
NEAA: Non-Essential Amino Acids
EF1α: Elongation Factor 1-alpha
CAGs: Cytomegalovirus early enhancer/chicken beta-actin promoter
HBSS: Hank's Balanced Salt Solution
HEPES: 4-(2-Hydroxyethyl)-1-piperazineethanesulfonic acid
*IFNT*: Interferon-tau
*MSTN*: Myostatin
IACUC: Institutional Animal Care and Use Committee
FACS: Fluorescence-Activated Cell Sorting
TALEN: Transcription Activator-Like Effector Nuclease
ZFN: Zinc-Finger Nuclease
PB: *PiggyBac*
OPU: Ovum Pick Up
SB: *Sleeping Beauty* (Transposon)
BSE: Bovine Spongiform Encephalopathy
cDNA: Complementary DNA
Pi: Injection Pressure
Pc: Constant Pressure
P/S: Penicillin/Streptomycin
mRNA: Messenger RNA
