# Supplementary material for: Cas9-expressing cattle using the PiggyBac transposon all-in-one system
Source: BMC Genomics. 2025 Mar 5;26:217. doi: 10.1186/s12864-025-11381-8 (PMC11881473; doi:10.1186/s12864-025-11381-8)
Supplement: Supplementary file 1 — Supplementary Material 1. [file 12864_2025_11381_MOESM1_ESM.zip › Supplemental Table S1.pptx]

## Slide 1
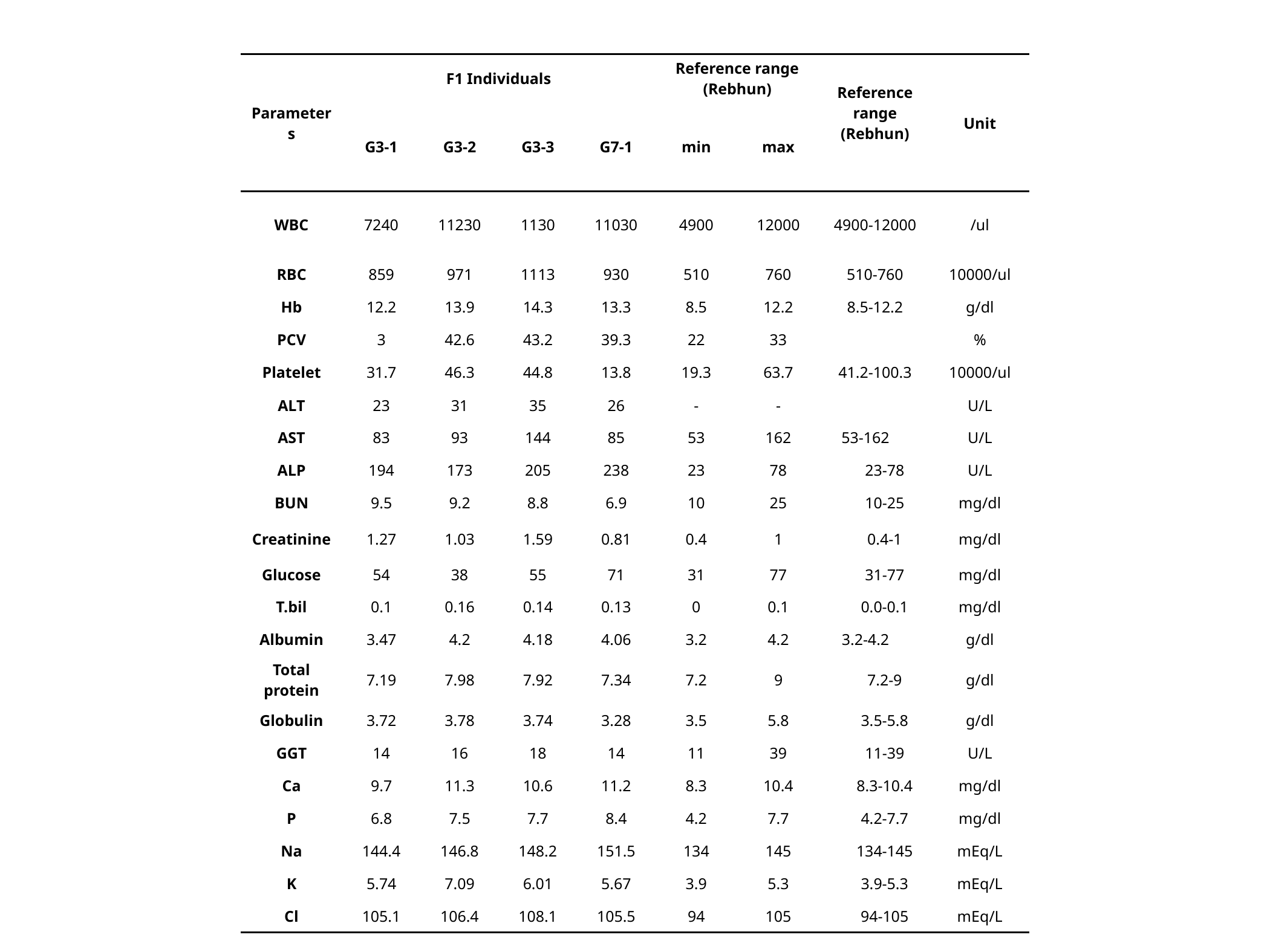

| Parameters | F1 Individuals | | | | Reference range (Rebhun) | | Reference range (Rebhun) | Unit |
| --- | --- | --- | --- | --- | --- | --- | --- | --- |
| | G3-1 | G3-2 | G3-3 | G7-1 | min | max | | |
| WBC | 7240 | 11230 | 1130 | 11030 | 4900 | 12000 | 4900-12000 | /ul |
| RBC | 859 | 971 | 1113 | 930 | 510 | 760 | 510-760 | 10000/ul |
| Hb | 12.2 | 13.9 | 14.3 | 13.3 | 8.5 | 12.2 | 8.5-12.2 | g/dl |
| PCV | 3 | 42.6 | 43.2 | 39.3 | 22 | 33 | | % |
| Platelet | 31.7 | 46.3 | 44.8 | 13.8 | 19.3 | 63.7 | 41.2-100.3 | 10000/ul |
| ALT | 23 | 31 | 35 | 26 | - | - | | U/L |
| AST | 83 | 93 | 144 | 85 | 53 | 162 | 53-162 | U/L |
| ALP | 194 | 173 | 205 | 238 | 23 | 78 | 23-78 | U/L |
| BUN | 9.5 | 9.2 | 8.8 | 6.9 | 10 | 25 | 10-25 | mg/dl |
| Creatinine | 1.27 | 1.03 | 1.59 | 0.81 | 0.4 | 1 | 0.4-1 | mg/dl |
| Glucose | 54 | 38 | 55 | 71 | 31 | 77 | 31-77 | mg/dl |
| T.bil | 0.1 | 0.16 | 0.14 | 0.13 | 0 | 0.1 | 0.0-0.1 | mg/dl |
| Albumin | 3.47 | 4.2 | 4.18 | 4.06 | 3.2 | 4.2 | 3.2-4.2 | g/dl |
| Total protein | 7.19 | 7.98 | 7.92 | 7.34 | 7.2 | 9 | 7.2-9 | g/dl |
| Globulin | 3.72 | 3.78 | 3.74 | 3.28 | 3.5 | 5.8 | 3.5-5.8 | g/dl |
| GGT | 14 | 16 | 18 | 14 | 11 | 39 | 11-39 | U/L |
| Ca | 9.7 | 11.3 | 10.6 | 11.2 | 8.3 | 10.4 | 8.3-10.4 | mg/dl |
| P | 6.8 | 7.5 | 7.7 | 8.4 | 4.2 | 7.7 | 4.2-7.7 | mg/dl |
| Na | 144.4 | 146.8 | 148.2 | 151.5 | 134 | 145 | 134-145 | mEq/L |
| K | 5.74 | 7.09 | 6.01 | 5.67 | 3.9 | 5.3 | 3.9-5.3 | mEq/L |
| Cl | 105.1 | 106.4 | 108.1 | 105.5 | 94 | 105 | 94-105 | mEq/L |
