# Supplementary material for: Cas9-expressing cattle using the PiggyBac transposon all-in-one system
Source: BMC Genomics. 2025 Mar 5;26:217. doi: 10.1186/s12864-025-11381-8 (PMC11881473; doi:10.1186/s12864-025-11381-8)
Supplement: Supplementary file 1 — Supplementary Material 1. [file 12864_2025_11381_MOESM1_ESM.zip › Supplemental Table S2.pptx]

## Slide 1
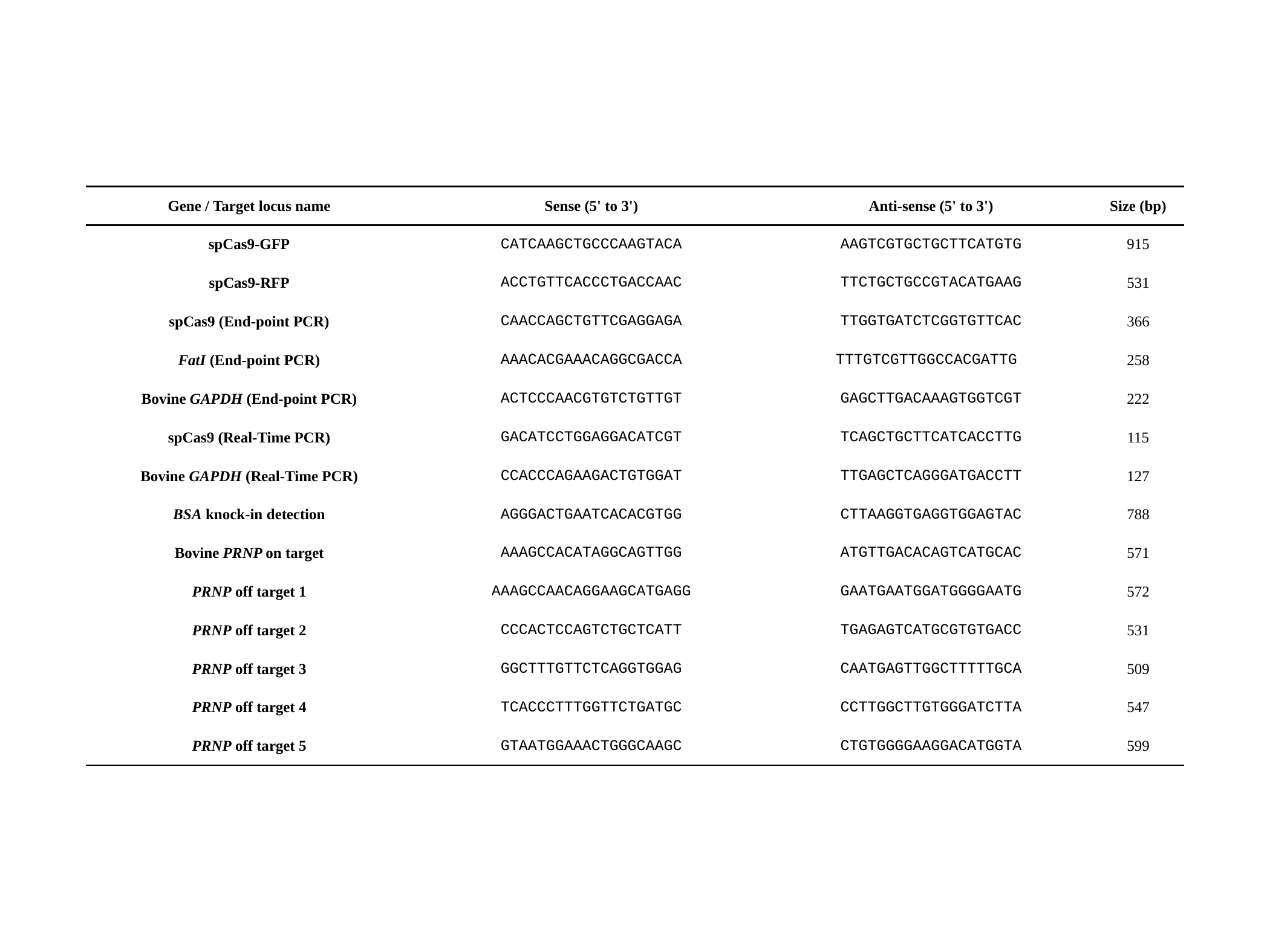

| Gene / Target locus name | Sense (5' to 3') | Anti-sense (5' to 3') | Size (bp) |
| --- | --- | --- | --- |
| spCas9-GFP | CATCAAGCTGCCCAAGTACA | AAGTCGTGCTGCTTCATGTG | 915 |
| spCas9-RFP | ACCTGTTCACCCTGACCAAC | TTCTGCTGCCGTACATGAAG | 531 |
| spCas9 (End-point PCR) | CAACCAGCTGTTCGAGGAGA | TTGGTGATCTCGGTGTTCAC | 366 |
| FatI (End-point PCR) | AAACACGAAACAGGCGACCA | TTTGTCGTTGGCCACGATTG | 258 |
| Bovine GAPDH (End-point PCR) | ACTCCCAACGTGTCTGTTGT | GAGCTTGACAAAGTGGTCGT | 222 |
| spCas9 (Real-Time PCR) | GACATCCTGGAGGACATCGT | TCAGCTGCTTCATCACCTTG | 115 |
| Bovine GAPDH (Real-Time PCR) | CCACCCAGAAGACTGTGGAT | TTGAGCTCAGGGATGACCTT | 127 |
| BSA knock-in detection | AGGGACTGAATCACACGTGG | CTTAAGGTGAGGTGGAGTAC | 788 |
| Bovine PRNP on target | AAAGCCACATAGGCAGTTGG | ATGTTGACACAGTCATGCAC | 571 |
| PRNP off target 1 | AAAGCCAACAGGAAGCATGAGG | GAATGAATGGATGGGGAATG | 572 |
| PRNP off target 2 | CCCACTCCAGTCTGCTCATT | TGAGAGTCATGCGTGTGACC | 531 |
| PRNP off target 3 | GGCTTTGTTCTCAGGTGGAG | CAATGAGTTGGCTTTTTGCA | 509 |
| PRNP off target 4 | TCACCCTTTGGTTCTGATGC | CCTTGGCTTGTGGGATCTTA | 547 |
| PRNP off target 5 | GTAATGGAAACTGGGCAAGC | CTGTGGGGAAGGACATGGTA | 599 |
