# Supplementary figures and images for: Cas9-expressing cattle using the PiggyBac transposon all-in-one system
Source: BMC Genomics. 2025 Mar 5;26:217. doi: 10.1186/s12864-025-11381-8 (PMC11881473; doi:10.1186/s12864-025-11381-8)

## Slide 1
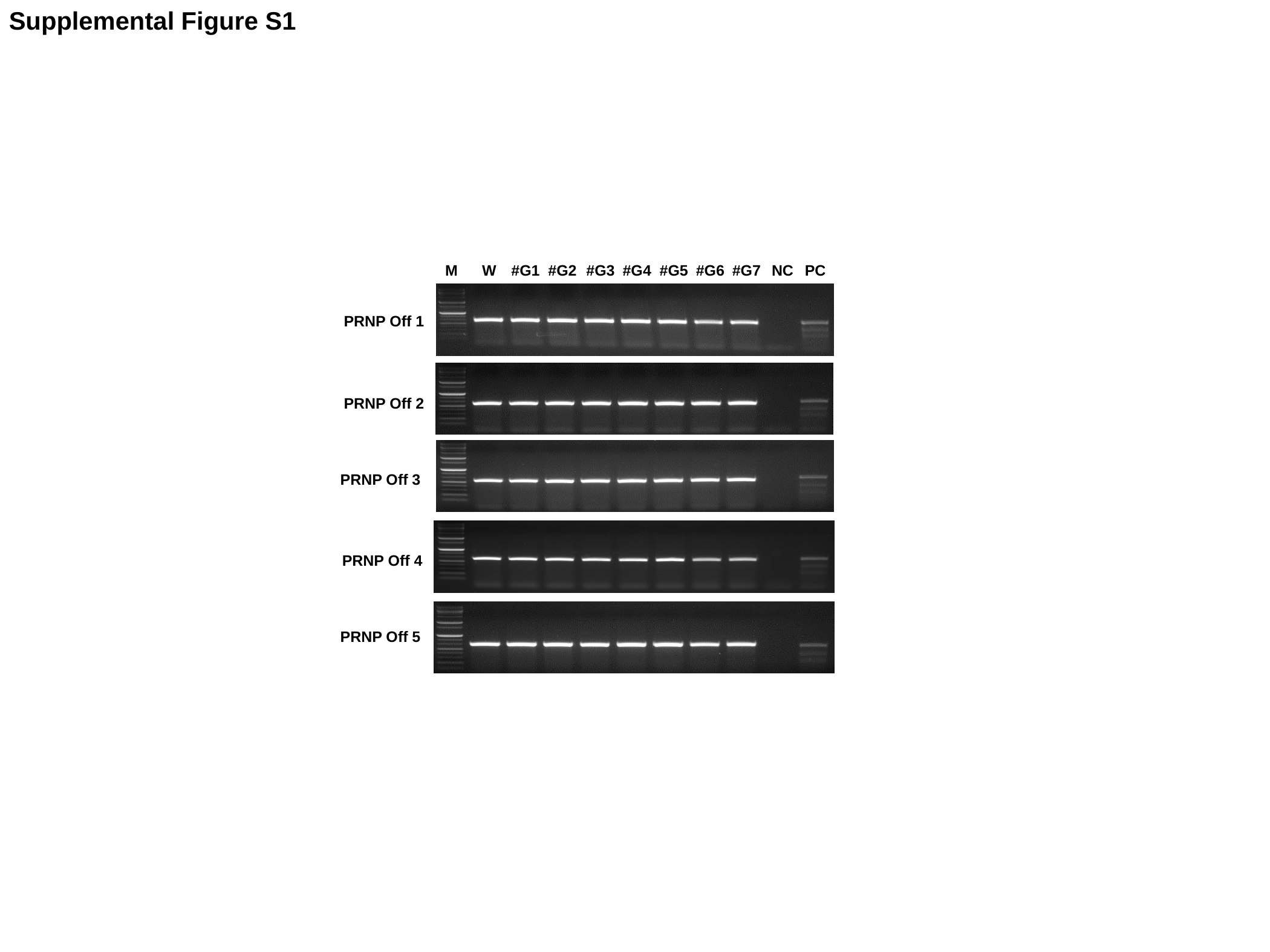

Supplemental Figure S1
M
W
#G1
#G2
#G3
#G4
#G5
#G6
#G7
NC
PC
PRNP Off 1
PRNP Off 2
PRNP Off 3
PRNP Off 4
PRNP Off 5

Supplement: Supplementary file 1 — Supplementary Material 1. [file 12864_2025_11381_MOESM1_ESM.zip › Supplemental Figure S1.pptx]

## Slide 1
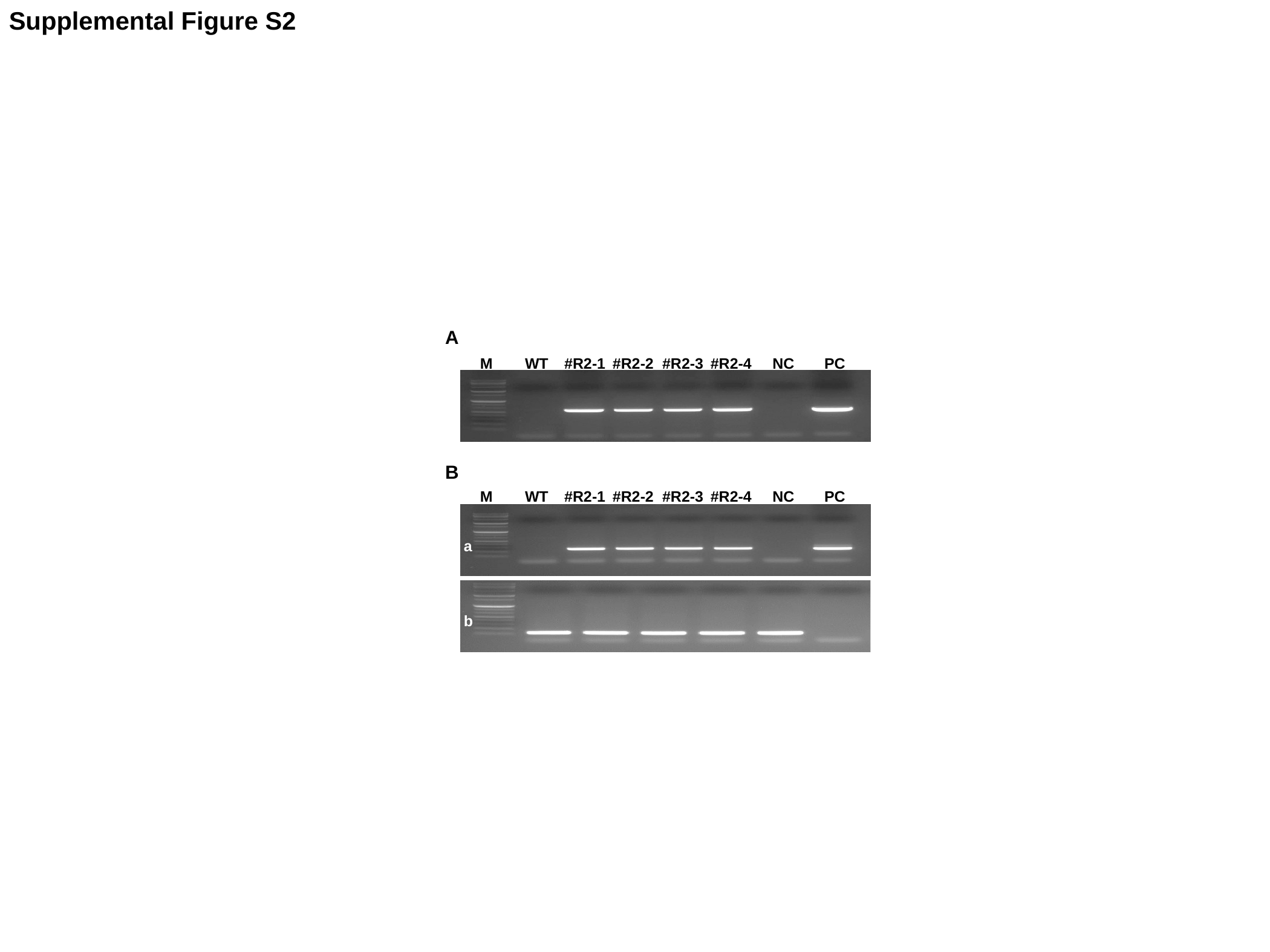

Supplemental Figure S2
A
M
WT
#R2-1
#R2-2
#R2-3
#R2-4
NC
PC
B
M
WT
#R2-1
#R2-2
#R2-3
#R2-4
NC
PC
a
b

Supplement: Supplementary file 1 — Supplementary Material 1. [file 12864_2025_11381_MOESM1_ESM.zip › Supplemental Figure S2.pptx]

## Slide 1
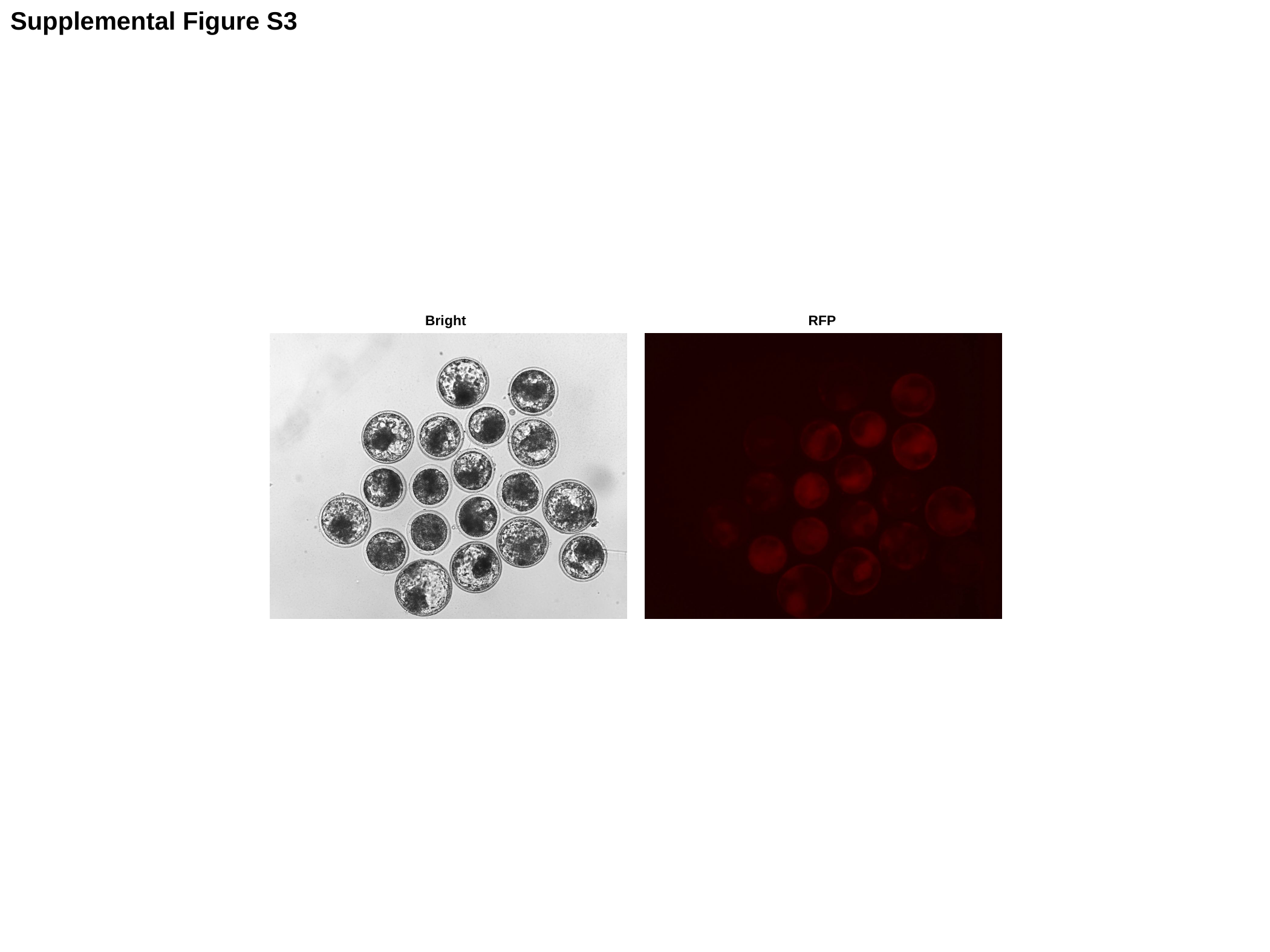

Supplemental Figure S3
RFP
Bright

Supplement: Supplementary file 1 — Supplementary Material 1. [file 12864_2025_11381_MOESM1_ESM.zip › Supplemental Figure S3.pptx]

## Slide 1
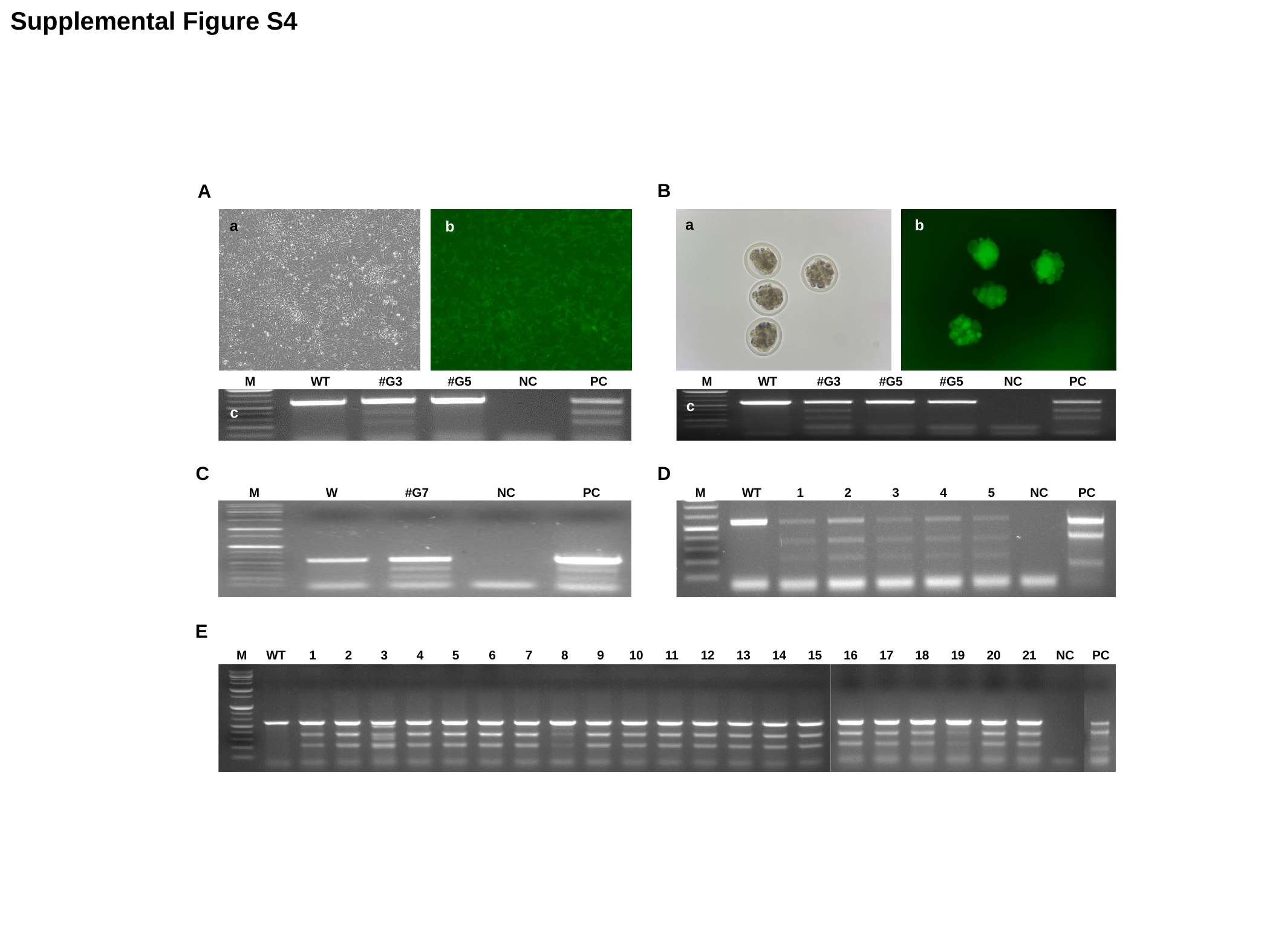

Supplemental Figure S4
B
A
a
b
a
b
M
WT
#G3
#G5
NC
PC
M
WT
#G3
#G5
#G5
NC
PC
c
c
C
D
M
W
#G7
NC
PC
M
WT
1
2
3
4
5
NC
PC
E
M
WT
1
2
3
4
5
6
7
8
9
10
11
12
13
14
15
16
17
18
19
20
21
NC
PC

Supplement: Supplementary file 1 — Supplementary Material 1. [file 12864_2025_11381_MOESM1_ESM.zip › Supplemental Figure S4.pptx]

## Slide 1
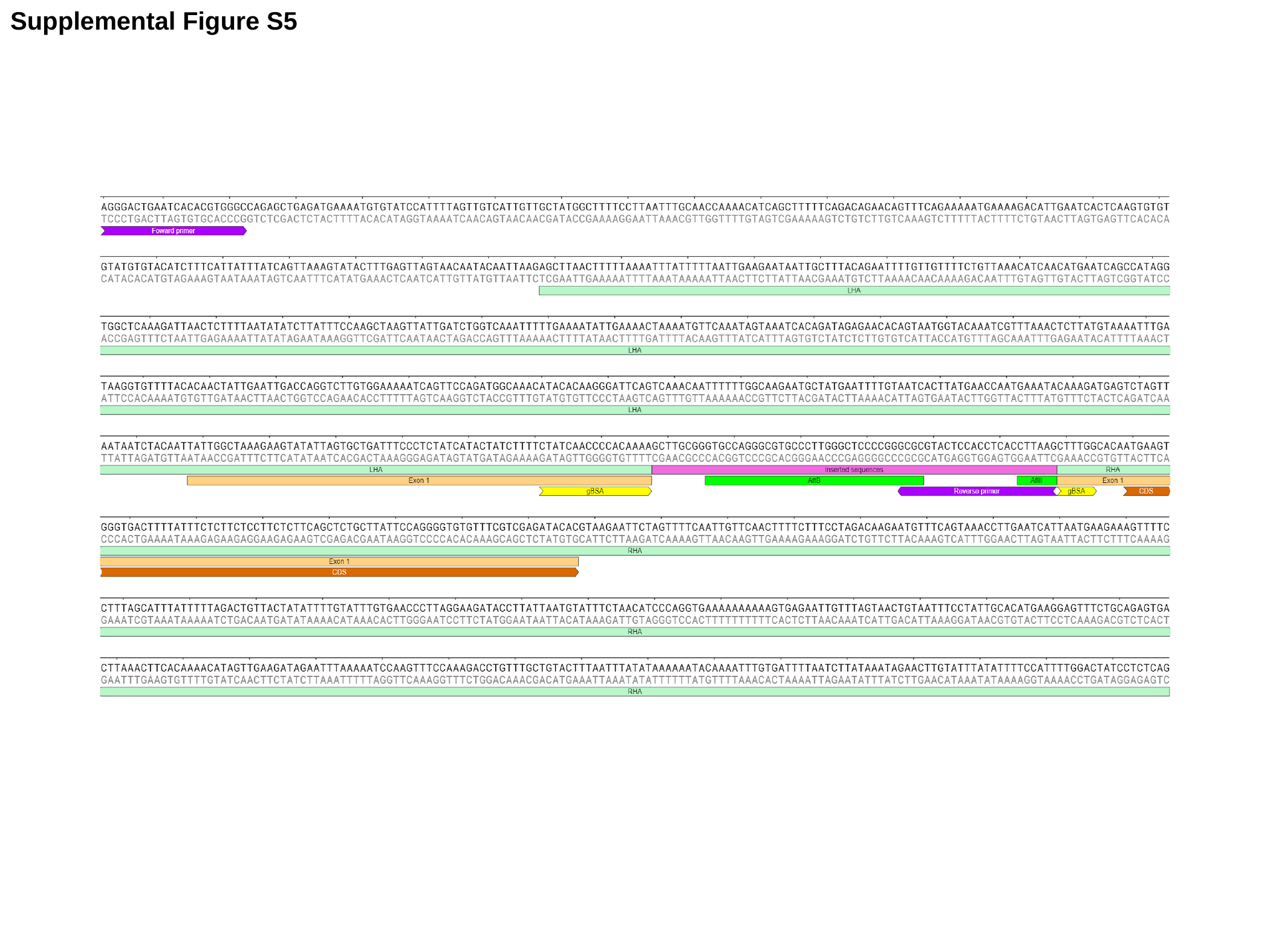

Supplemental Figure S5

Supplement: Supplementary file 1 — Supplementary Material 1. [file 12864_2025_11381_MOESM1_ESM.zip › Supplemental Figure S5.pptx]

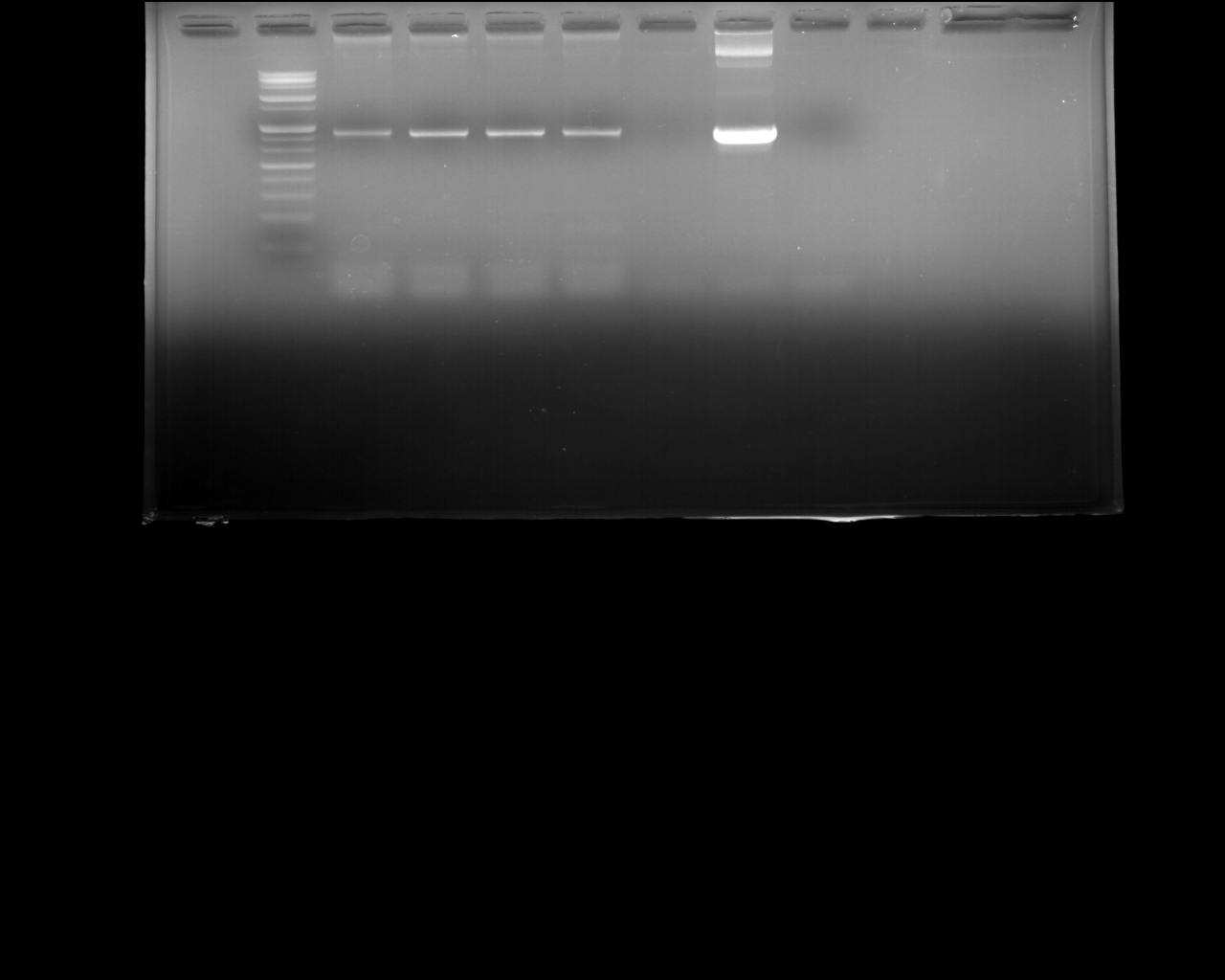

Supplement: Supplementary file 2 — Supplementary Material 2. [file 12864_2025_11381_MOESM2_ESM.zip › Supplementary file (Original gels and blots)/Figure 1/Figure 1C-a.tif]

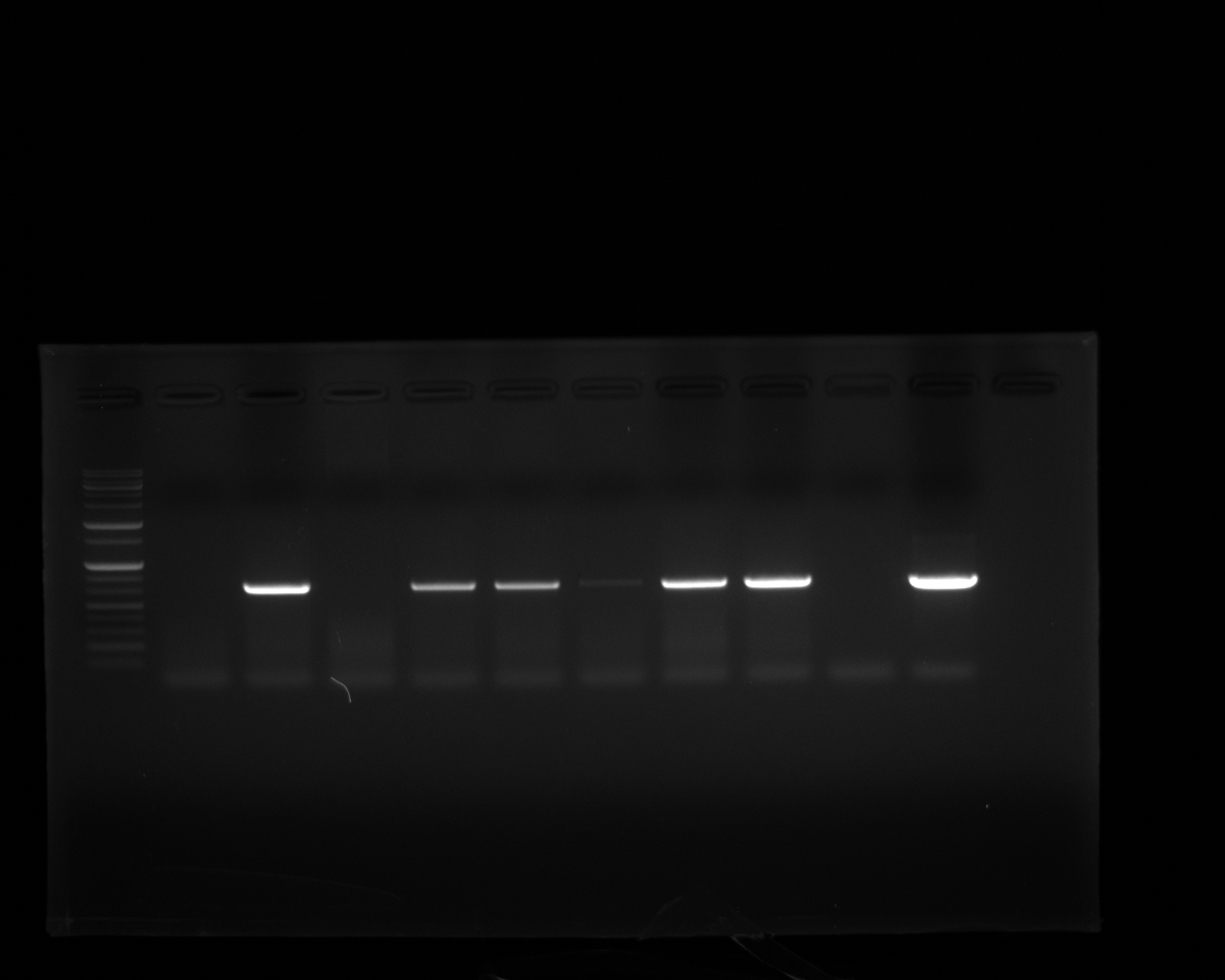

Supplement: Supplementary file 2 — Supplementary Material 2. [file 12864_2025_11381_MOESM2_ESM.zip › Supplementary file (Original gels and blots)/Figure 1/Figure 1C-b.tif]

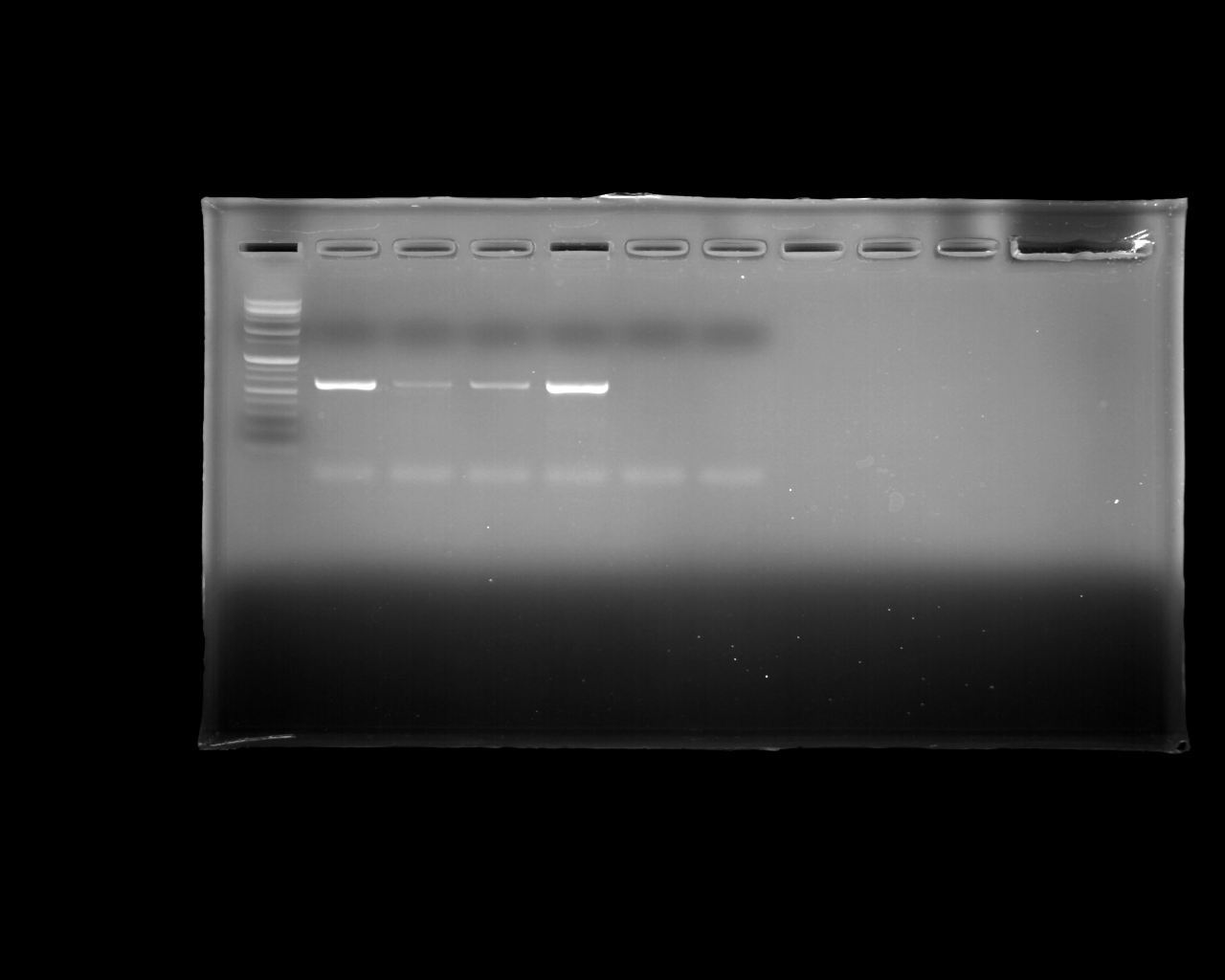

Supplement: Supplementary file 2 — Supplementary Material 2. [file 12864_2025_11381_MOESM2_ESM.zip › Supplementary file (Original gels and blots)/Figure 1/Figure 1D-a.tif]

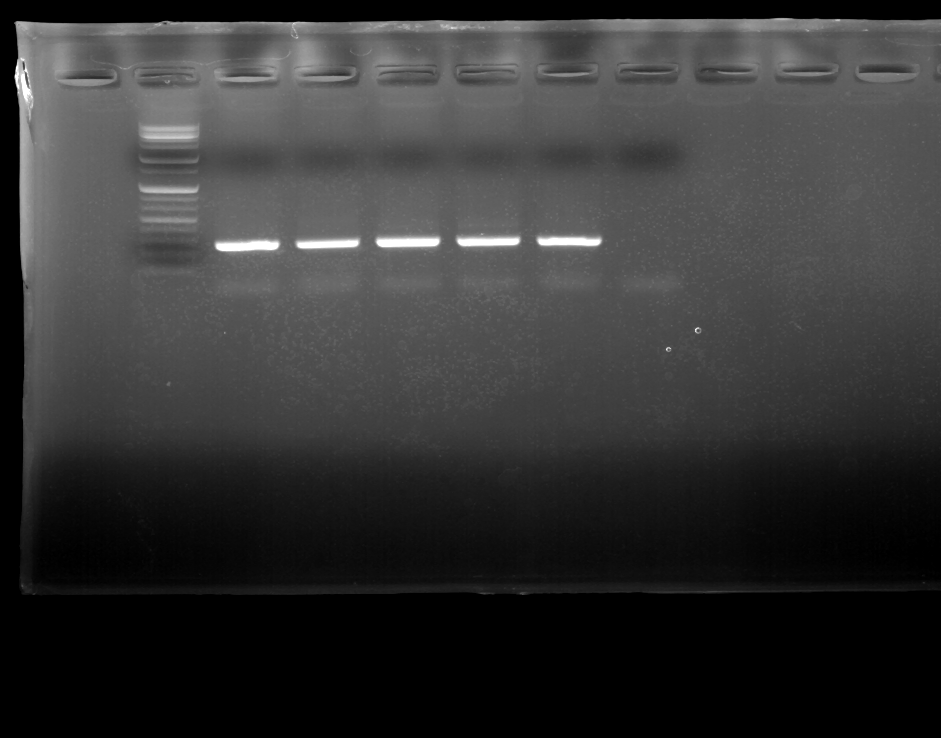

Supplement: Supplementary file 2 — Supplementary Material 2. [file 12864_2025_11381_MOESM2_ESM.zip › Supplementary file (Original gels and blots)/Figure 1/Figure 1D-b.tif]

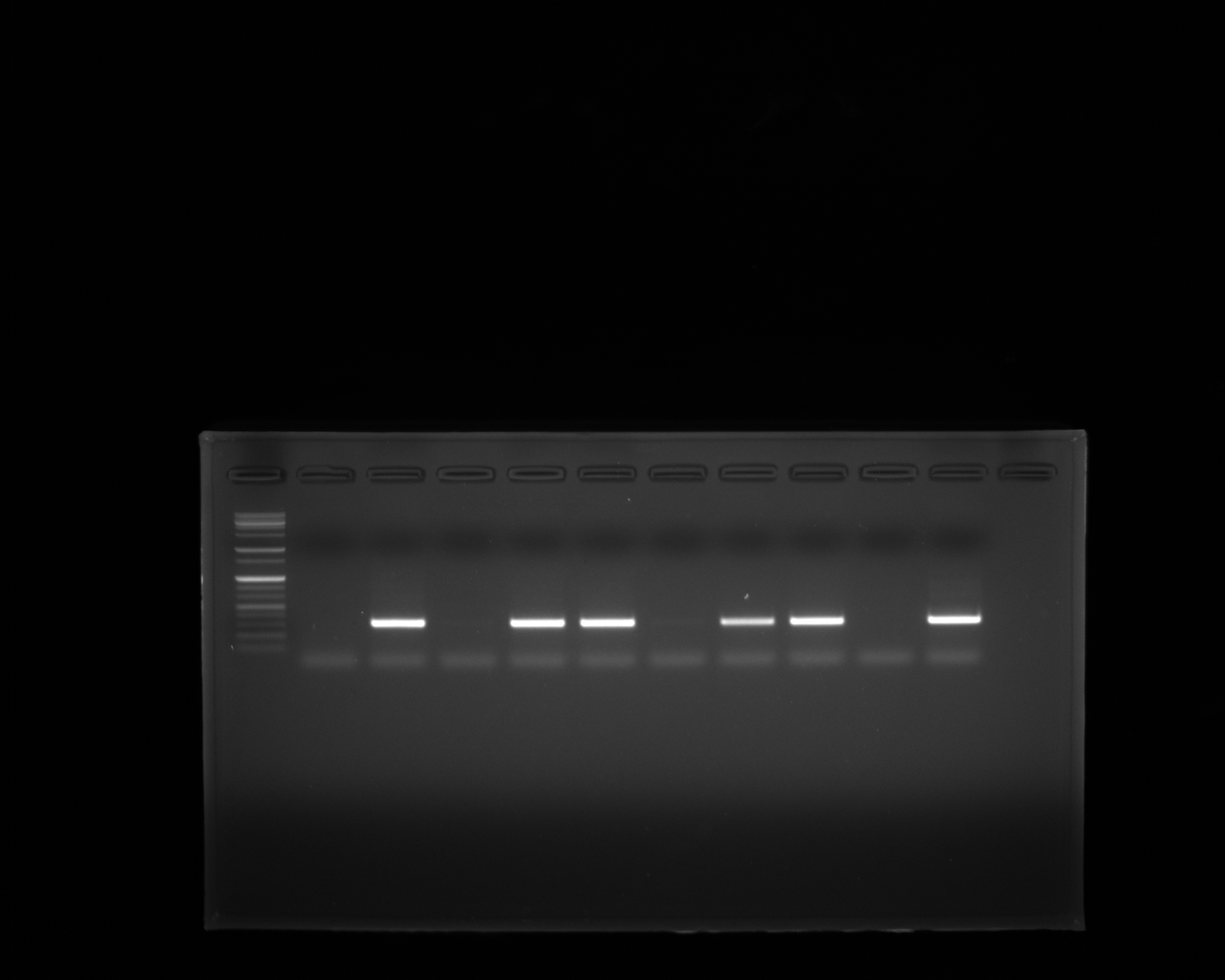

Supplement: Supplementary file 2 — Supplementary Material 2. [file 12864_2025_11381_MOESM2_ESM.zip › Supplementary file (Original gels and blots)/Figure 1/Figure 1D-c.tif]

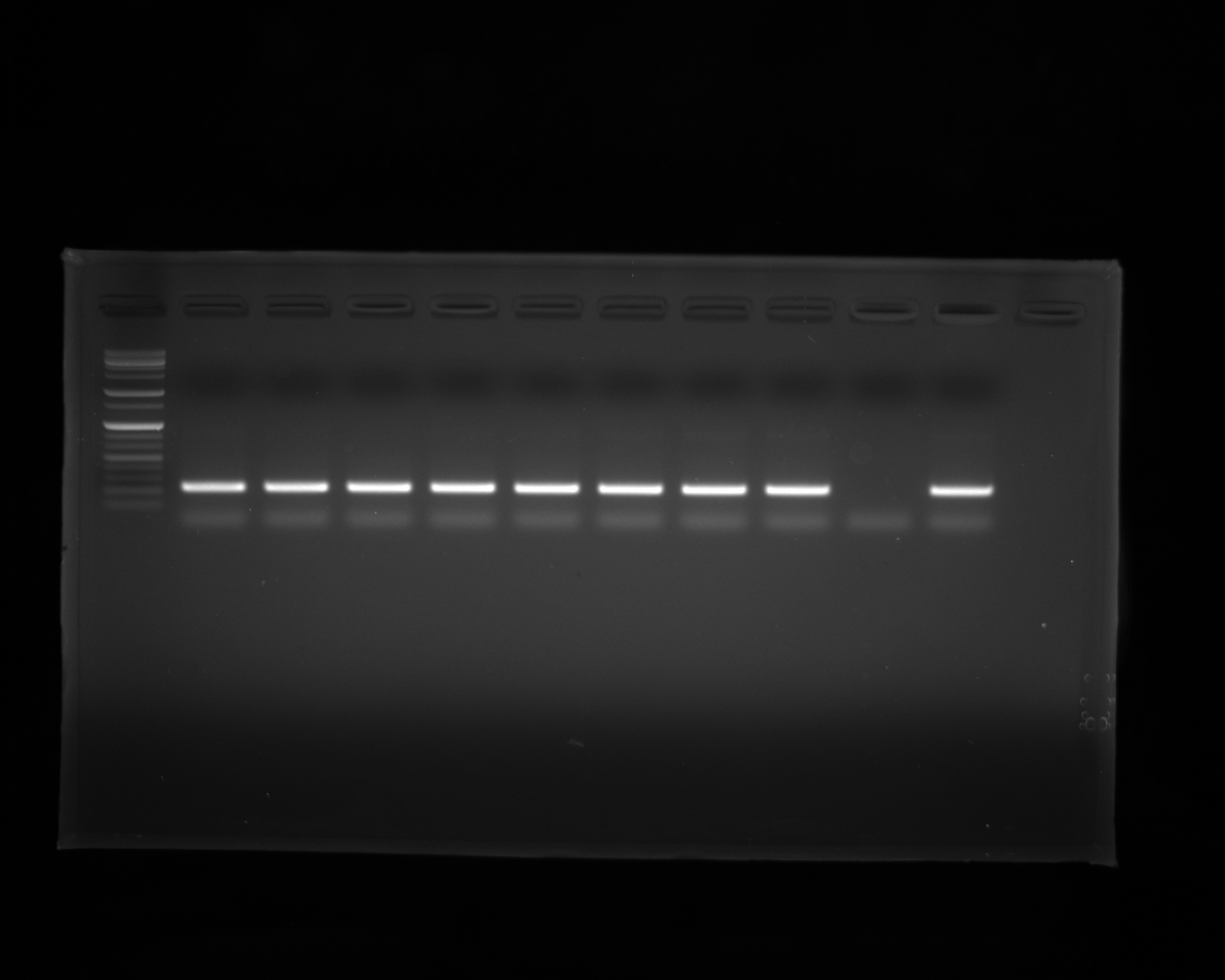

Supplement: Supplementary file 2 — Supplementary Material 2. [file 12864_2025_11381_MOESM2_ESM.zip › Supplementary file (Original gels and blots)/Figure 1/Figure 1D-d.tif]

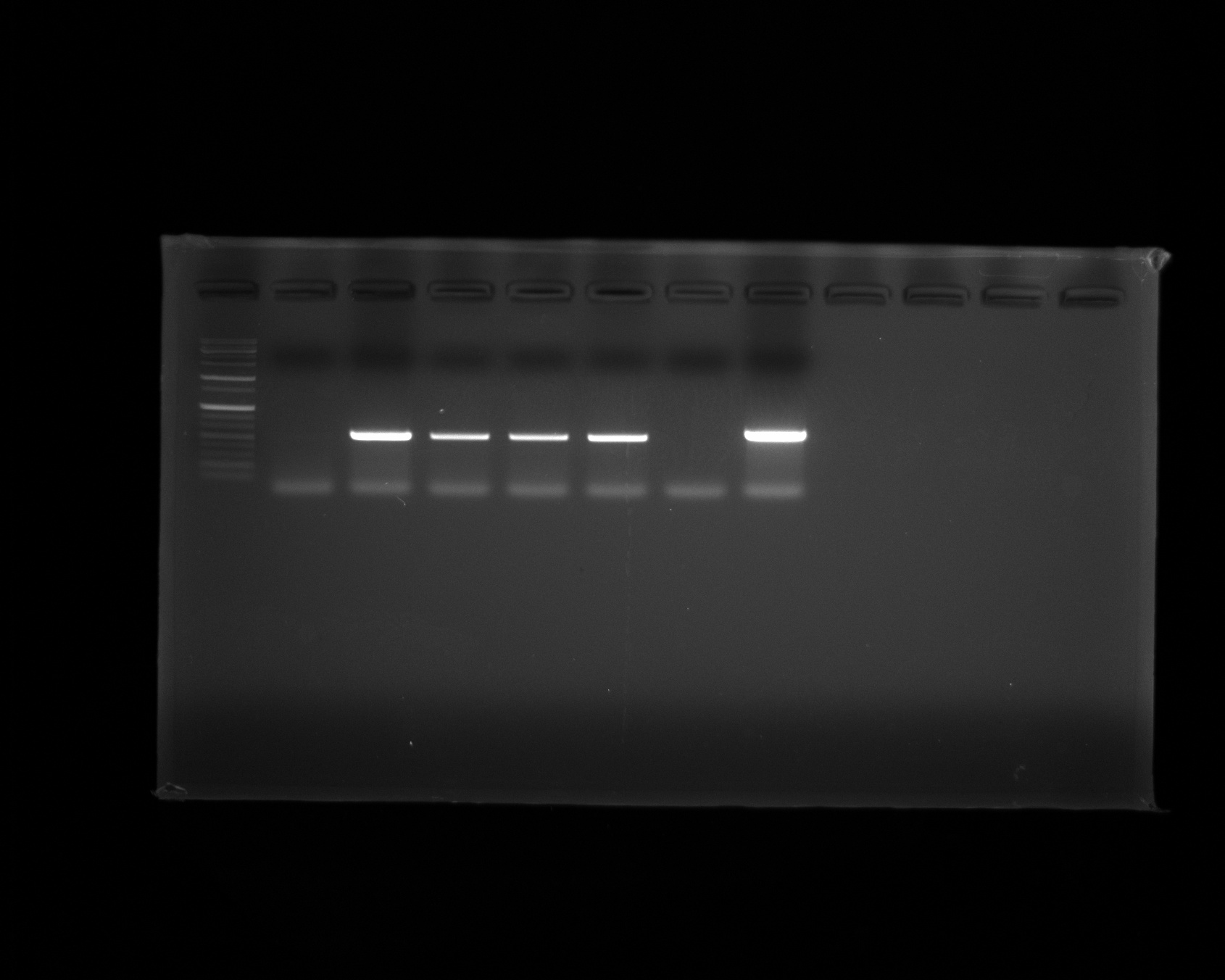

Supplement: Supplementary file 2 — Supplementary Material 2. [file 12864_2025_11381_MOESM2_ESM.zip › Supplementary file (Original gels and blots)/Figure 2/Figure 2C.jpg]

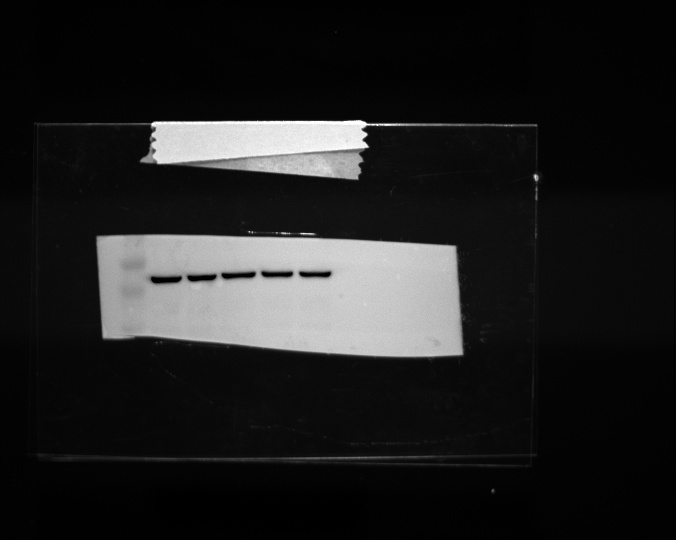

Supplement: Supplementary file 2 — Supplementary Material 2. [file 12864_2025_11381_MOESM2_ESM.zip › Supplementary file (Original gels and blots)/Figure 2/Figure 2D-Beta-Actin.jpg]

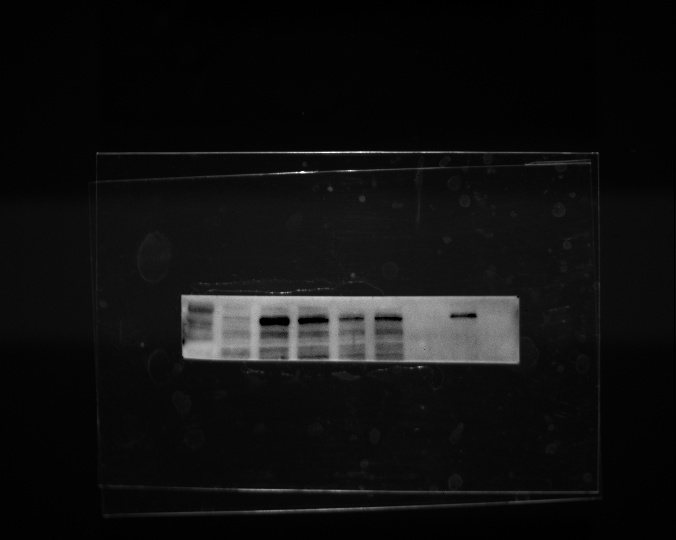

Supplement: Supplementary file 2 — Supplementary Material 2. [file 12864_2025_11381_MOESM2_ESM.zip › Supplementary file (Original gels and blots)/Figure 2/Figure 2D-Cas9-Merged.jpg]

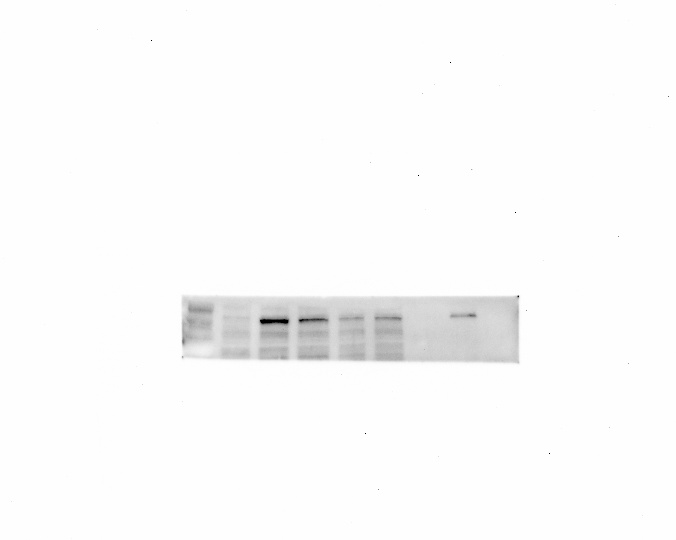

Supplement: Supplementary file 2 — Supplementary Material 2. [file 12864_2025_11381_MOESM2_ESM.zip › Supplementary file (Original gels and blots)/Figure 2/Figure 2D-Cas9.jpg]

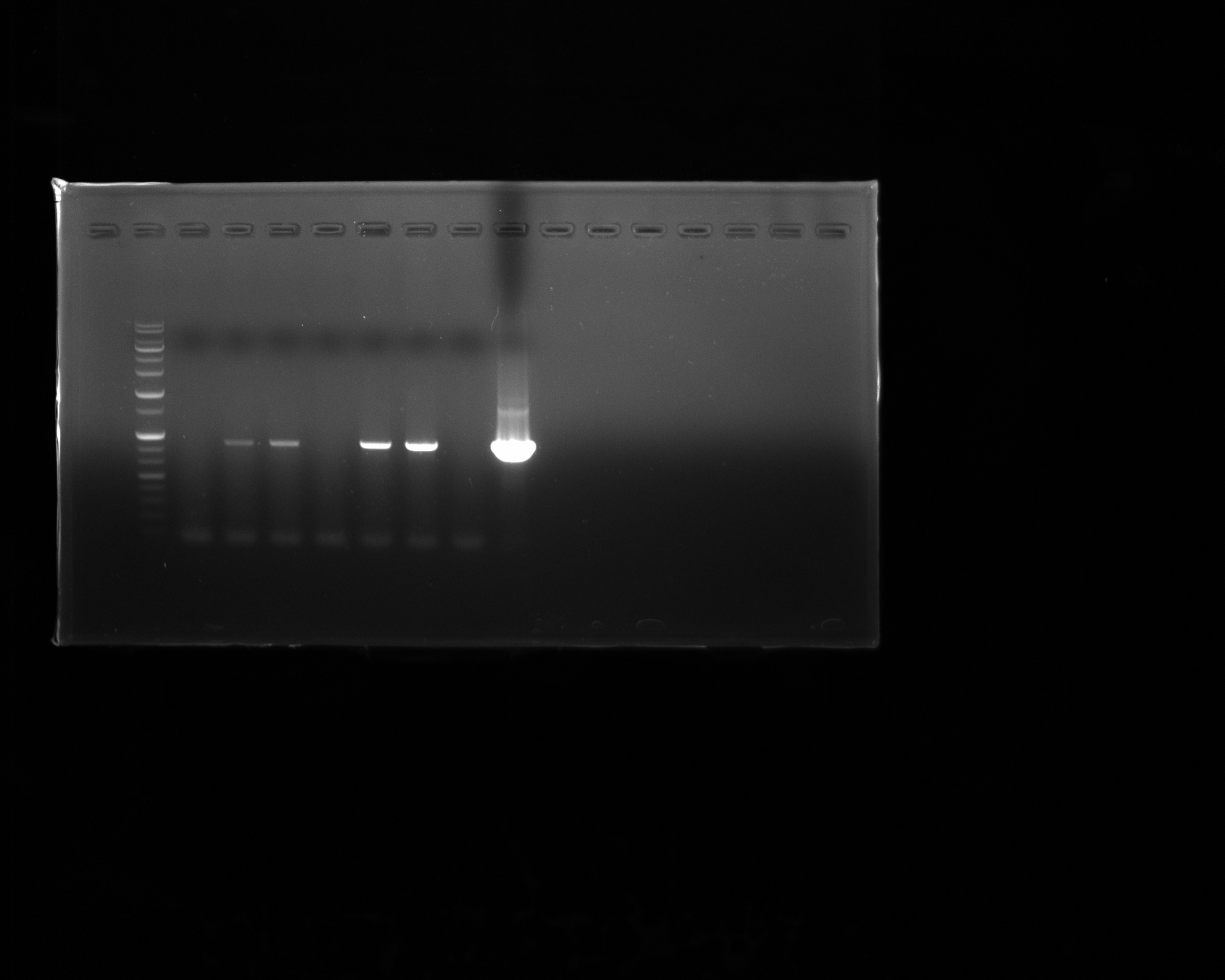

Supplement: Supplementary file 2 — Supplementary Material 2. [file 12864_2025_11381_MOESM2_ESM.zip › Supplementary file (Original gels and blots)/Figure 3/Figure 3B.jpg]

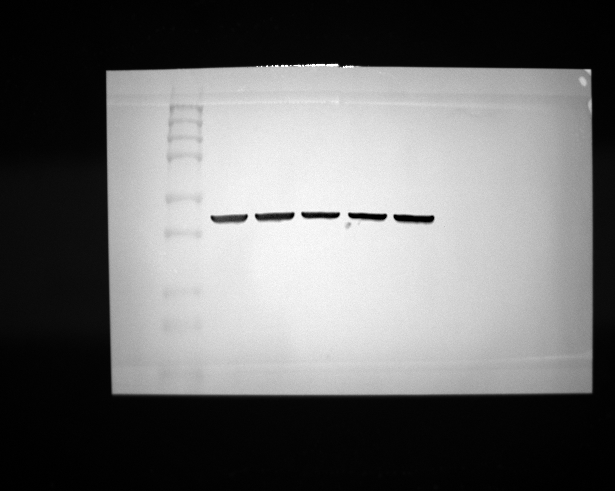

Supplement: Supplementary file 2 — Supplementary Material 2. [file 12864_2025_11381_MOESM2_ESM.zip › Supplementary file (Original gels and blots)/Figure 3/Figure 3C-Beta-Actin.tif]

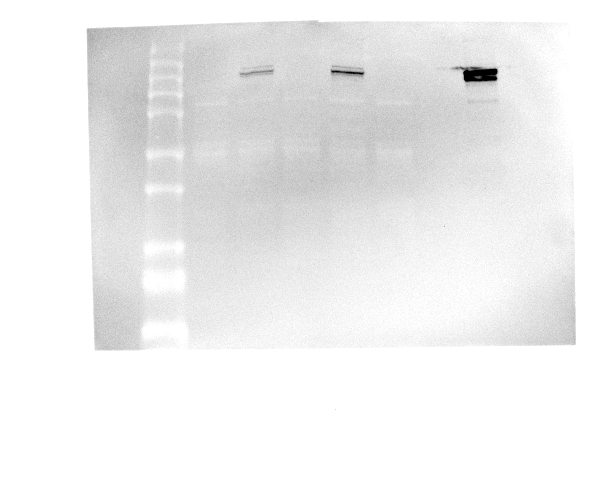

Supplement: Supplementary file 2 — Supplementary Material 2. [file 12864_2025_11381_MOESM2_ESM.zip › Supplementary file (Original gels and blots)/Figure 3/Figure 3C-Cas9.tif]

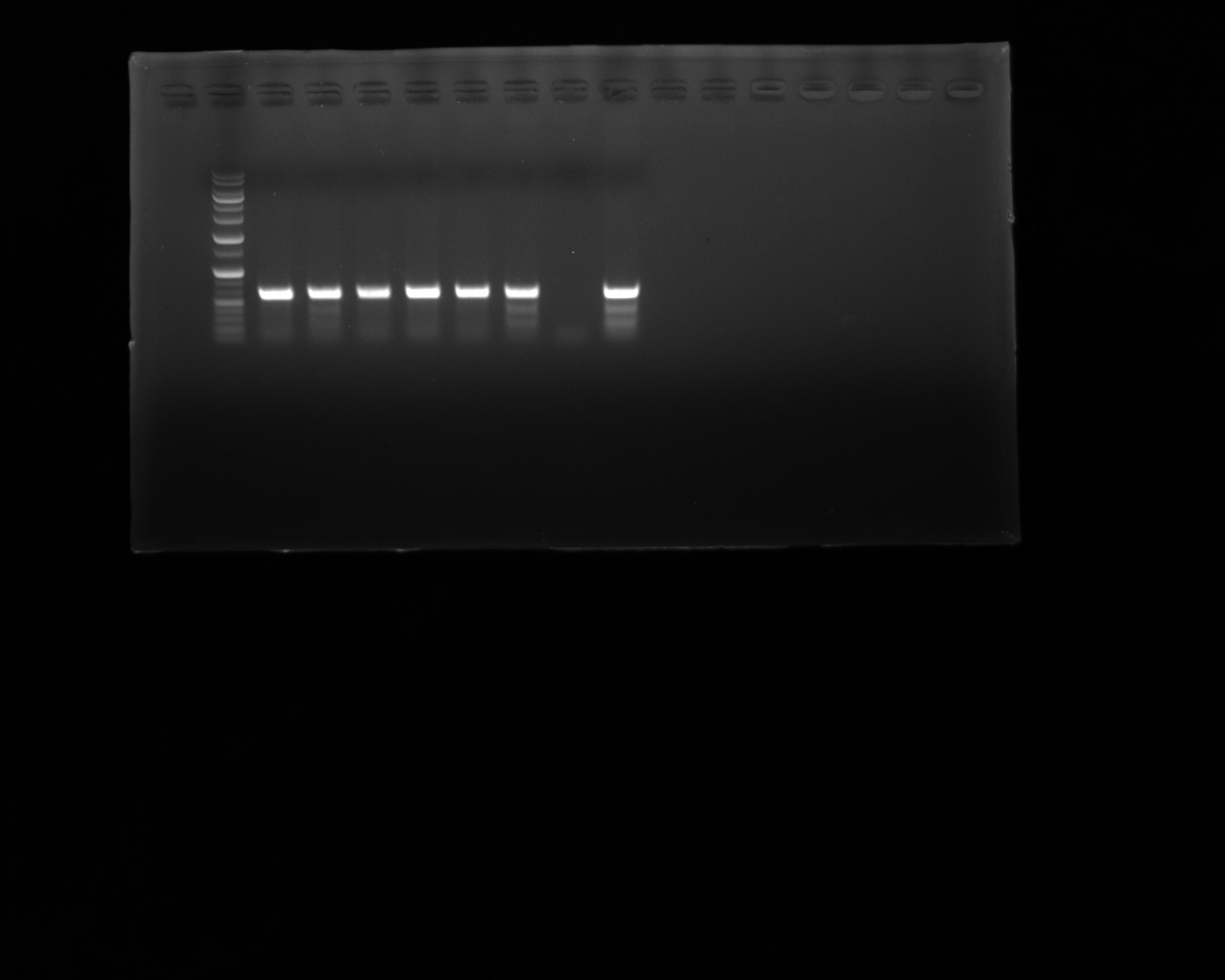

Supplement: Supplementary file 2 — Supplementary Material 2. [file 12864_2025_11381_MOESM2_ESM.zip › Supplementary file (Original gels and blots)/Figure 3/Figure 3D.jpg]

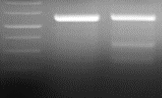

Supplement: Supplementary file 2 — Supplementary Material 2. [file 12864_2025_11381_MOESM2_ESM.zip › Supplementary file (Original gels and blots)/Figure 4/Figure 4A-BCN(+T7E1).png]

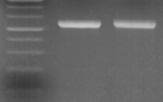

Supplement: Supplementary file 2 — Supplementary Material 2. [file 12864_2025_11381_MOESM2_ESM.zip › Supplementary file (Original gels and blots)/Figure 4/Figure 4A-BCN(-T7E1).png]

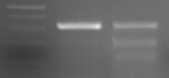

Supplement: Supplementary file 2 — Supplementary Material 2. [file 12864_2025_11381_MOESM2_ESM.zip › Supplementary file (Original gels and blots)/Figure 4/Figure 4A-BLG(+T7E1).png]

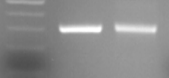

Supplement: Supplementary file 2 — Supplementary Material 2. [file 12864_2025_11381_MOESM2_ESM.zip › Supplementary file (Original gels and blots)/Figure 4/Figure 4A-BLG(-T7E1).png]

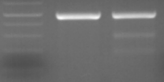

Supplement: Supplementary file 2 — Supplementary Material 2. [file 12864_2025_11381_MOESM2_ESM.zip › Supplementary file (Original gels and blots)/Figure 4/Figure 4A-Nanog(+T7E1).png]

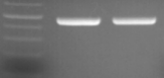

Supplement: Supplementary file 2 — Supplementary Material 2. [file 12864_2025_11381_MOESM2_ESM.zip › Supplementary file (Original gels and blots)/Figure 4/Figure 4A-Nanog(-T7E1).png]

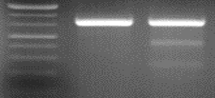

Supplement: Supplementary file 2 — Supplementary Material 2. [file 12864_2025_11381_MOESM2_ESM.zip › Supplementary file (Original gels and blots)/Figure 4/Figure 4A-P53(+T7E1).png]

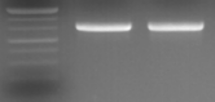

Supplement: Supplementary file 2 — Supplementary Material 2. [file 12864_2025_11381_MOESM2_ESM.zip › Supplementary file (Original gels and blots)/Figure 4/Figure 4A-P53(-T7E1).png]

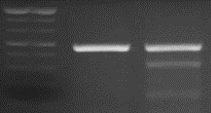

Supplement: Supplementary file 2 — Supplementary Material 2. [file 12864_2025_11381_MOESM2_ESM.zip › Supplementary file (Original gels and blots)/Figure 4/Figure 4A-PrnP(+T7E1).png]

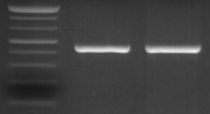

Supplement: Supplementary file 2 — Supplementary Material 2. [file 12864_2025_11381_MOESM2_ESM.zip › Supplementary file (Original gels and blots)/Figure 4/Figure 4A-PrnP(-T7E1).png]

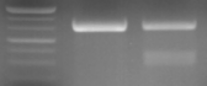

Supplement: Supplementary file 2 — Supplementary Material 2. [file 12864_2025_11381_MOESM2_ESM.zip › Supplementary file (Original gels and blots)/Figure 4/Figure 4A-Rb1(+T7E1).png]

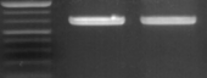

Supplement: Supplementary file 2 — Supplementary Material 2. [file 12864_2025_11381_MOESM2_ESM.zip › Supplementary file (Original gels and blots)/Figure 4/Figure 4A-Rb1(-T7E1).png]

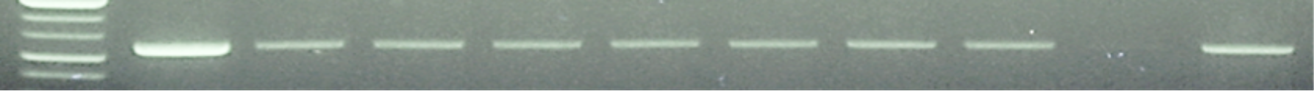

Supplement: Supplementary file 2 — Supplementary Material 2. [file 12864_2025_11381_MOESM2_ESM.zip › Supplementary file (Original gels and blots)/Figure 4/Figure 4D-PCR.png]

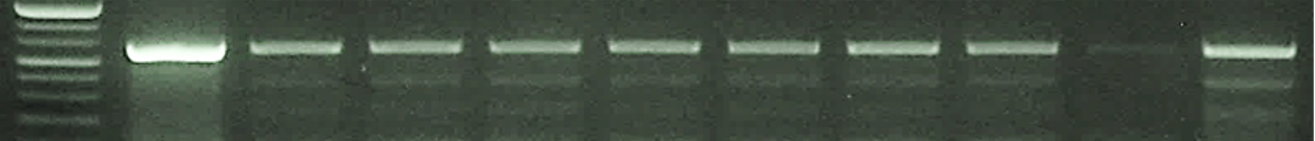

Supplement: Supplementary file 2 — Supplementary Material 2. [file 12864_2025_11381_MOESM2_ESM.zip › Supplementary file (Original gels and blots)/Figure 4/Figure 4D-T7E1.png]

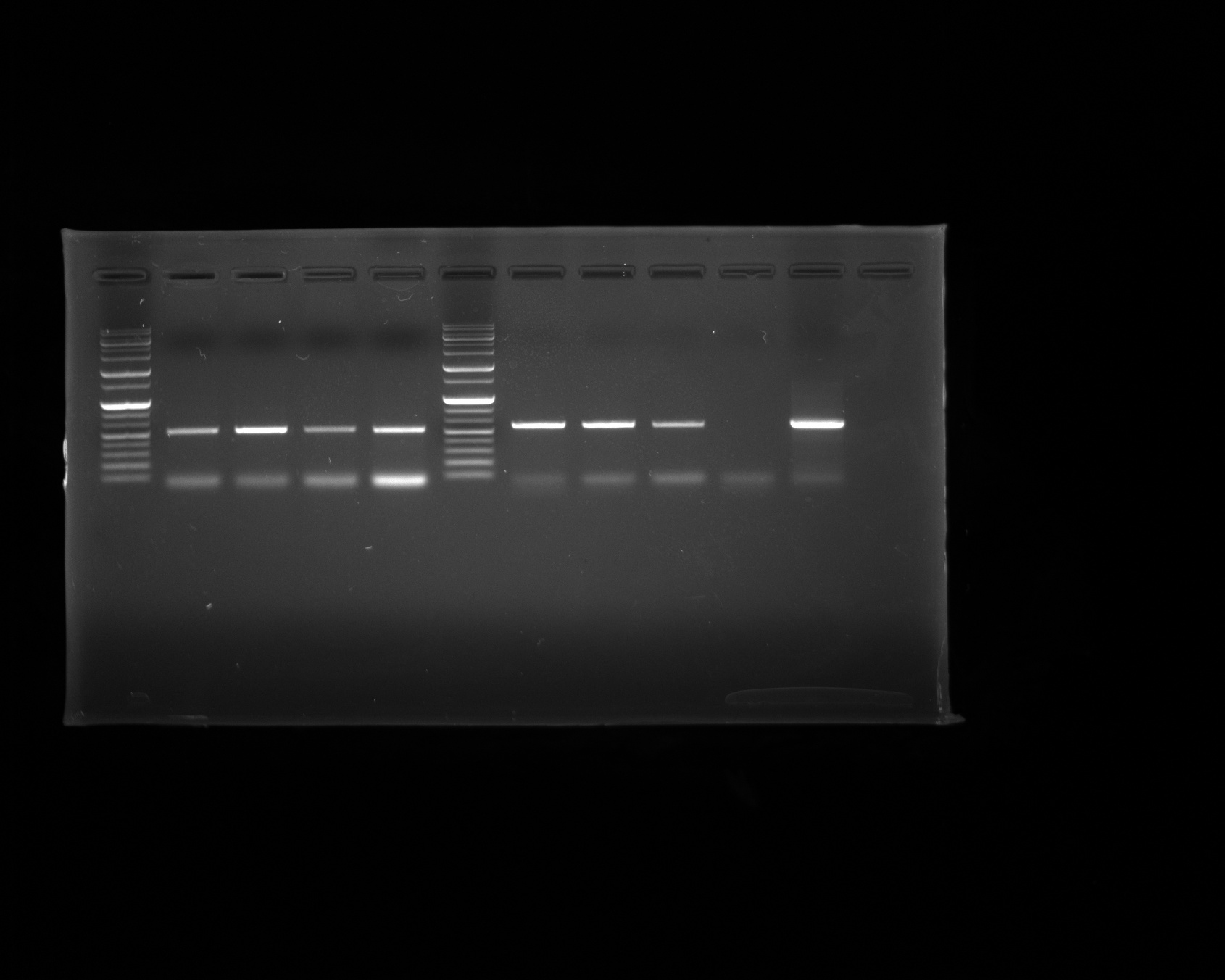

Supplement: Supplementary file 2 — Supplementary Material 2. [file 12864_2025_11381_MOESM2_ESM.zip › Supplementary file (Original gels and blots)/Figure 5/Figure 5C_PCR (Right side gel, from marker to 5th well).jpg]

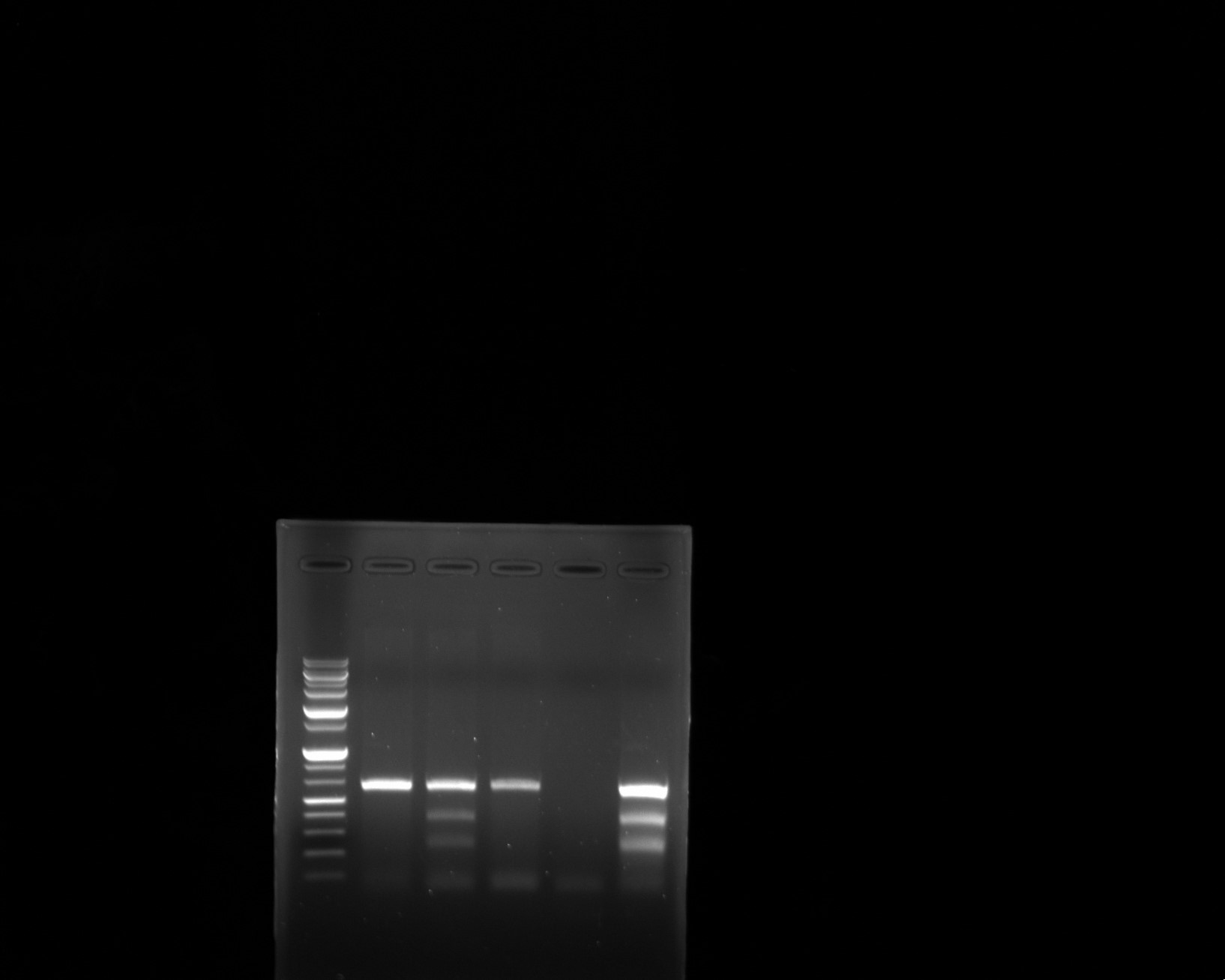

Supplement: Supplementary file 2 — Supplementary Material 2. [file 12864_2025_11381_MOESM2_ESM.zip › Supplementary file (Original gels and blots)/Figure 5/Figure 5C_T7E1.jpg]

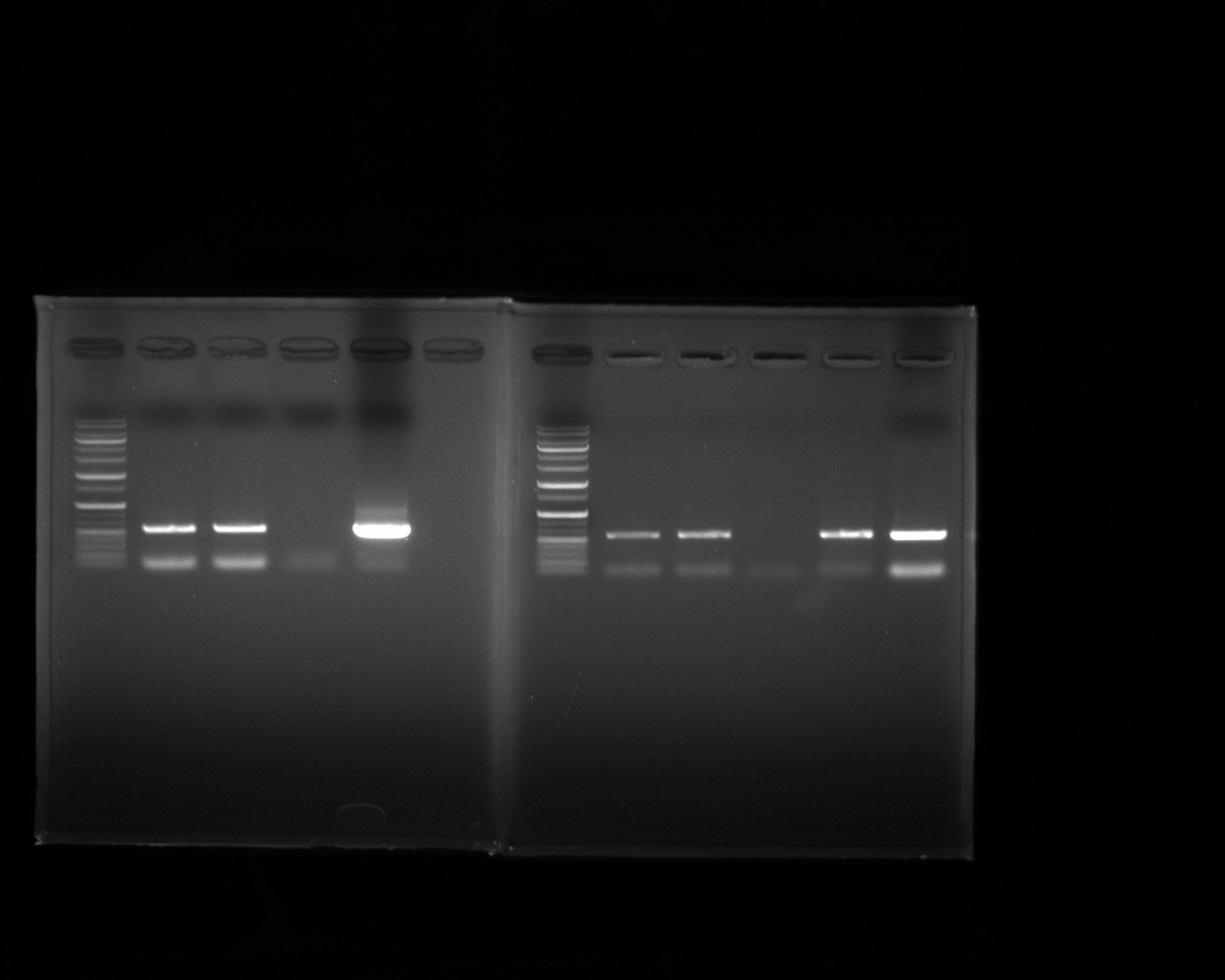

Supplement: Supplementary file 2 — Supplementary Material 2. [file 12864_2025_11381_MOESM2_ESM.zip › Supplementary file (Original gels and blots)/Figure 5/Figure 5E_PCR (Right side gel, from marker to 4th well).jpg]

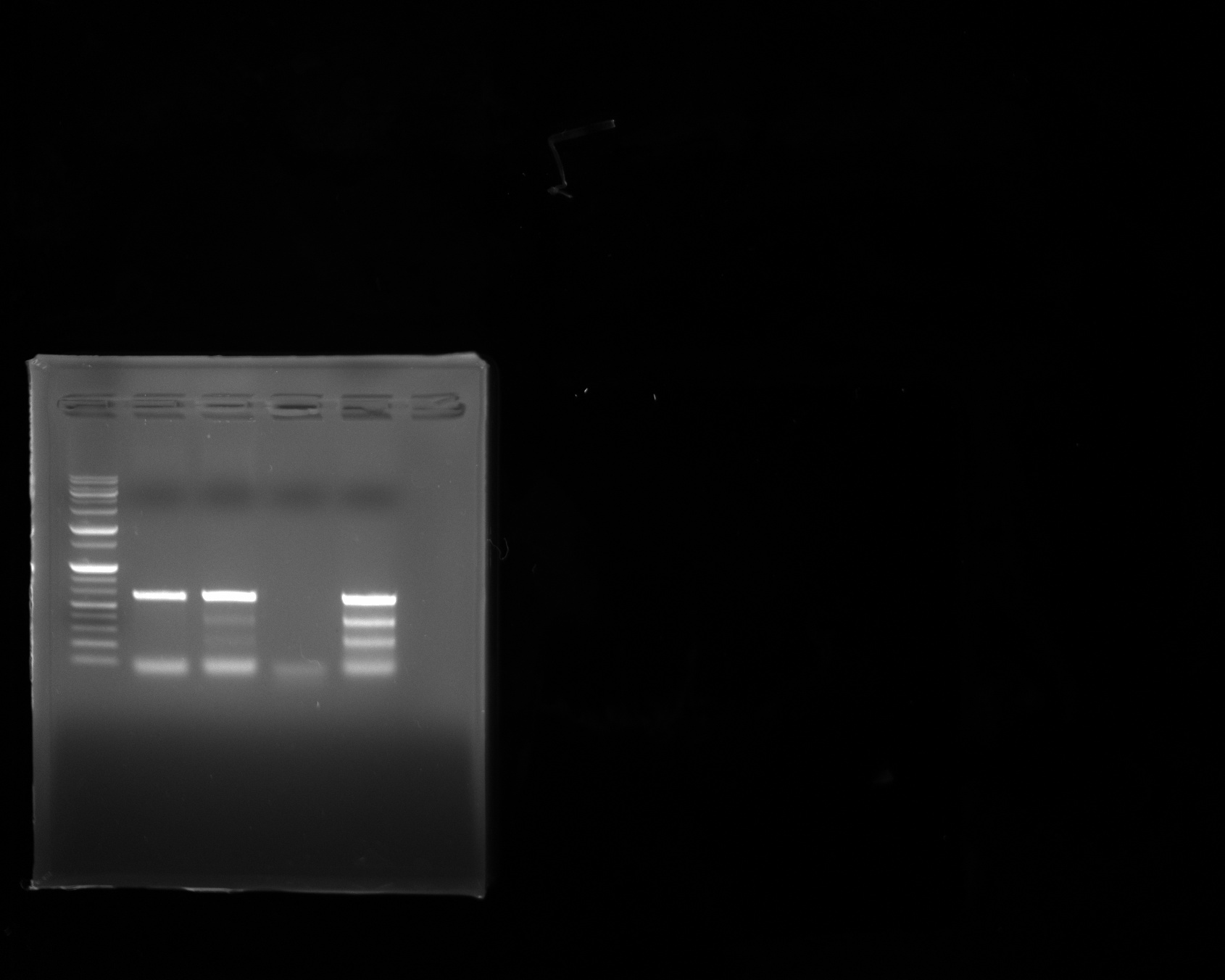

Supplement: Supplementary file 2 — Supplementary Material 2. [file 12864_2025_11381_MOESM2_ESM.zip › Supplementary file (Original gels and blots)/Figure 5/Figure 5E_T7E1.jpg]

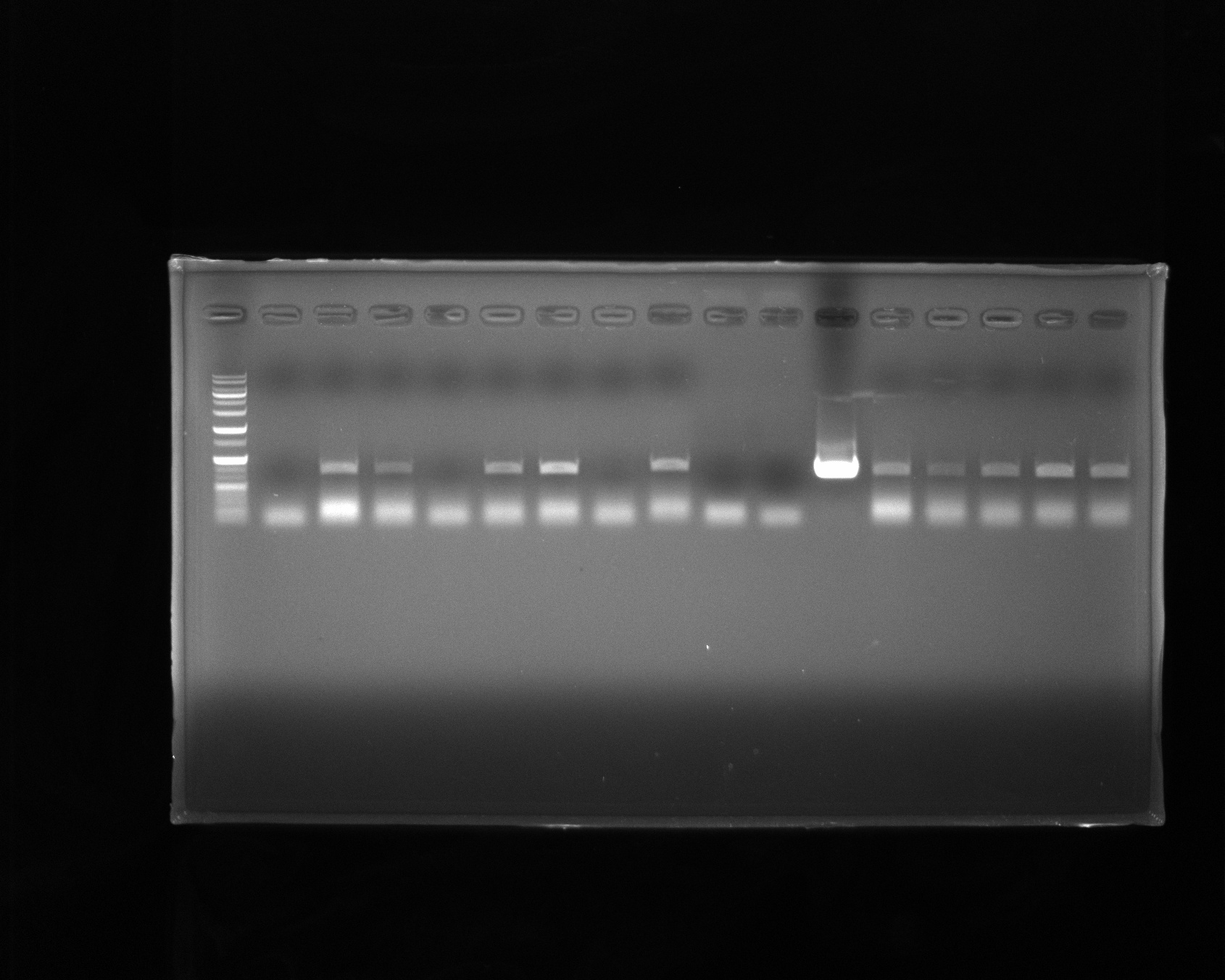

Supplement: Supplementary file 2 — Supplementary Material 2. [file 12864_2025_11381_MOESM2_ESM.zip › Supplementary file (Original gels and blots)/Figure 5/Figure 5G_b_PCR (from marker to 10th well).jpg]

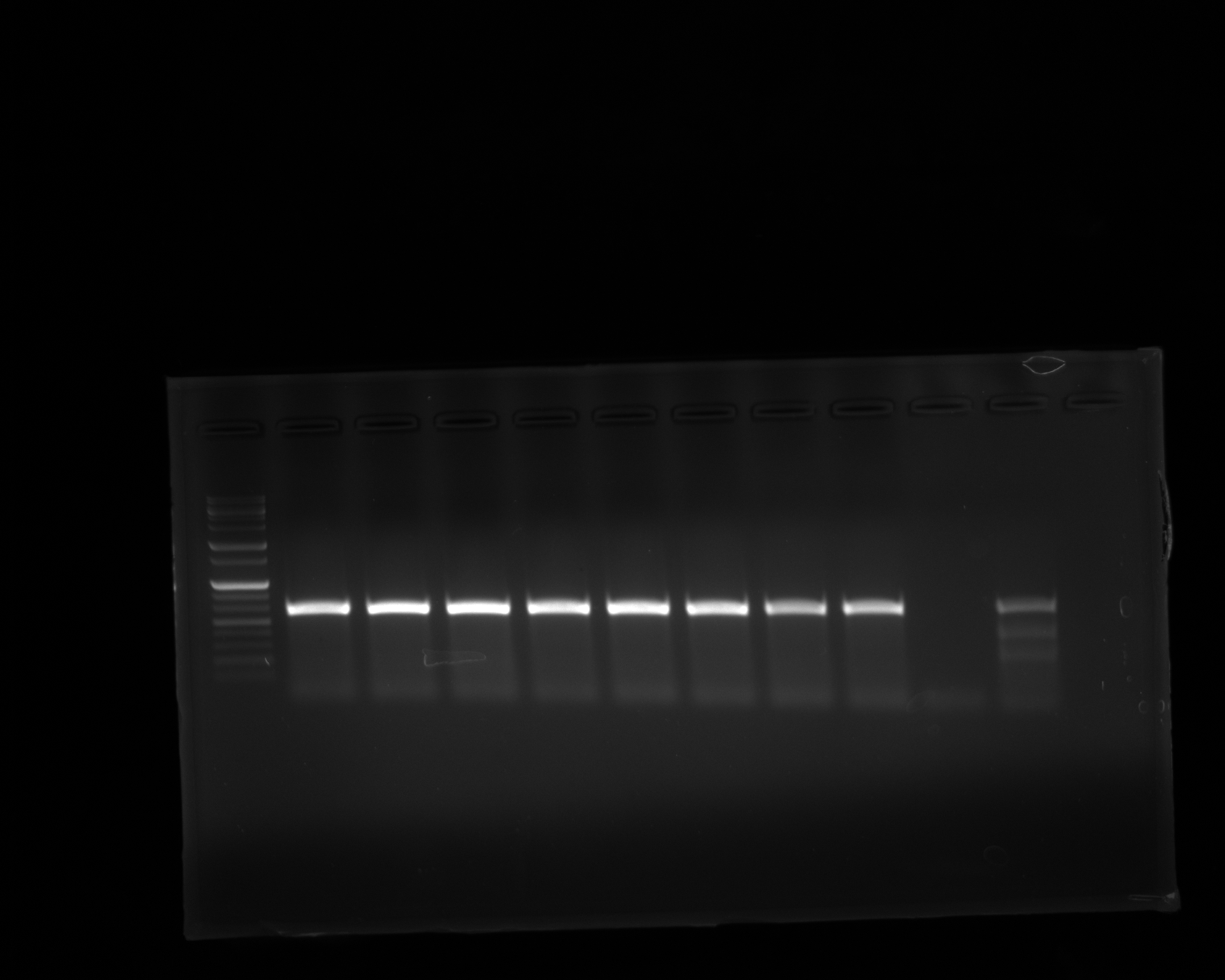

Supplement: Supplementary file 2 — Supplementary Material 2. [file 12864_2025_11381_MOESM2_ESM.zip › Supplementary file (Original gels and blots)/Supplementary Figure 1/PRNP off1.tif]

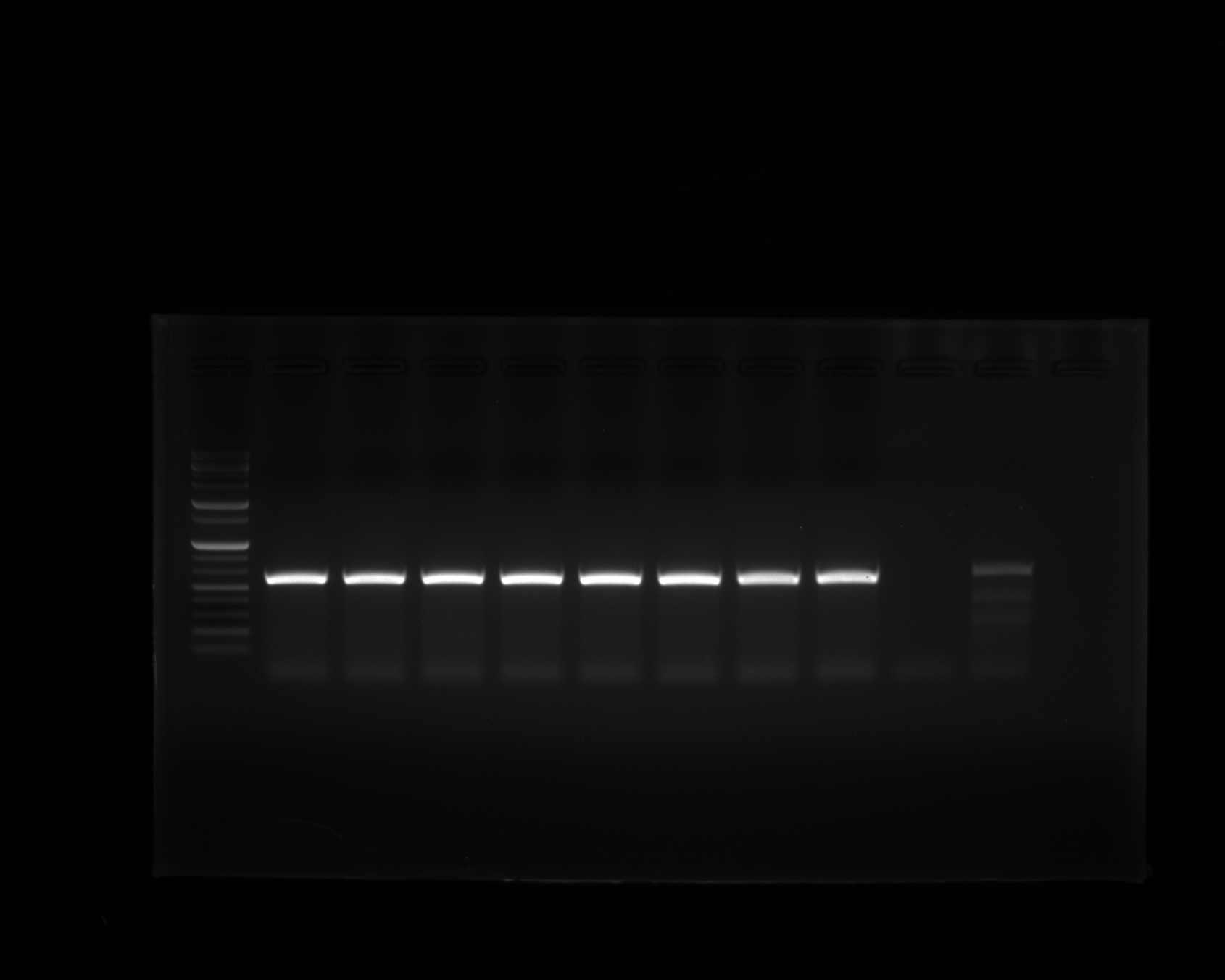

Supplement: Supplementary file 2 — Supplementary Material 2. [file 12864_2025_11381_MOESM2_ESM.zip › Supplementary file (Original gels and blots)/Supplementary Figure 1/PRNP off2.tif]

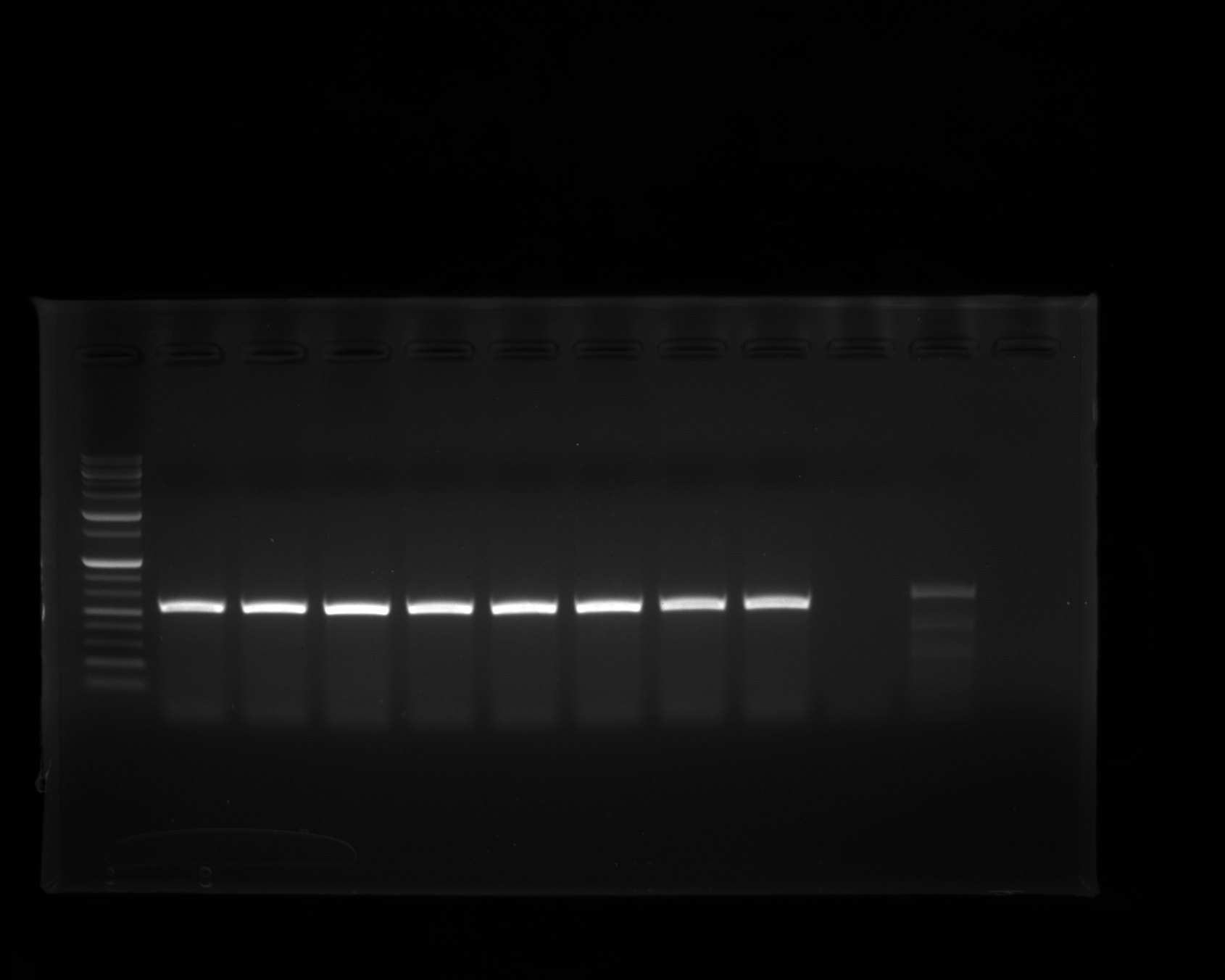

Supplement: Supplementary file 2 — Supplementary Material 2. [file 12864_2025_11381_MOESM2_ESM.zip › Supplementary file (Original gels and blots)/Supplementary Figure 1/PRNP off3.tif]

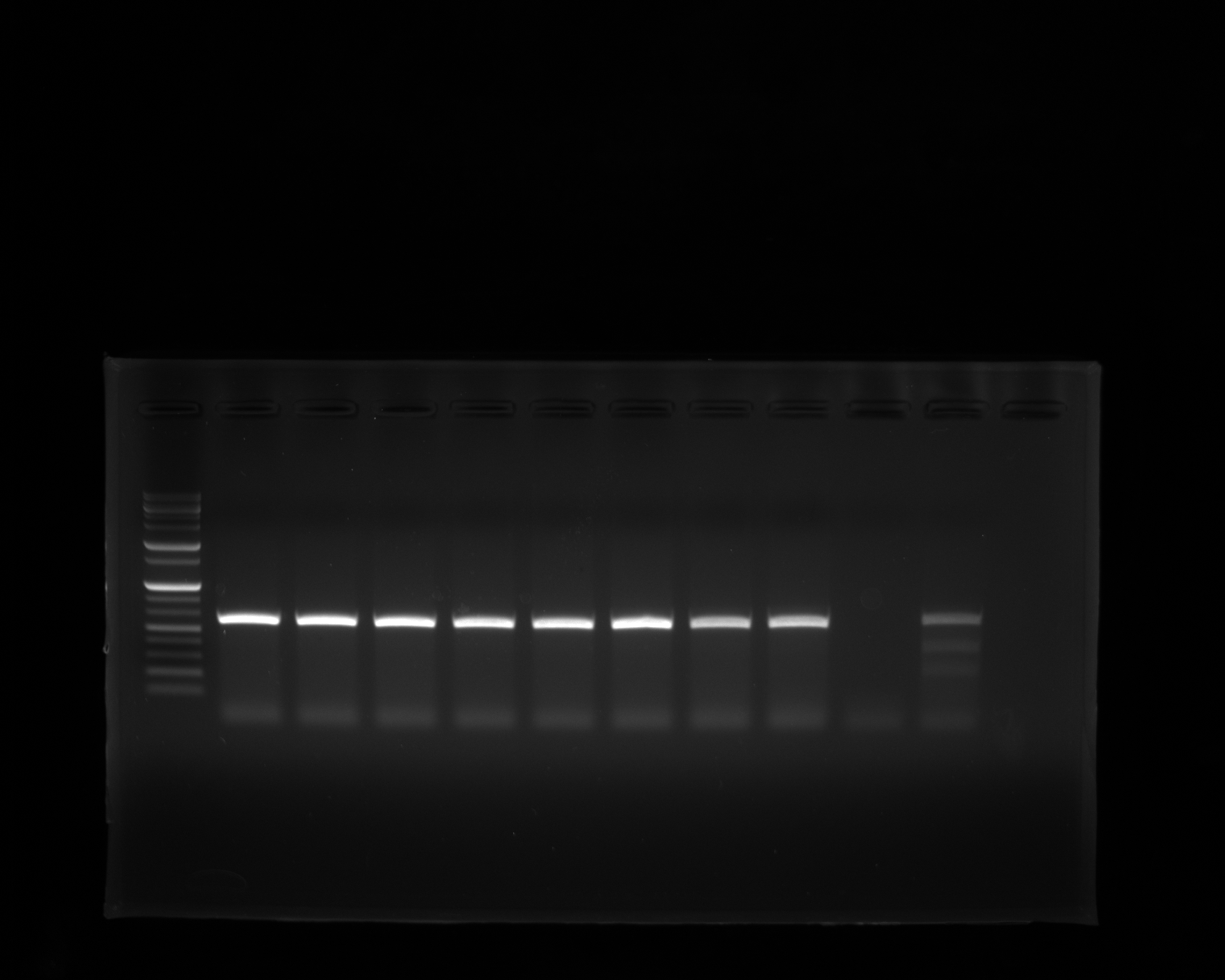

Supplement: Supplementary file 2 — Supplementary Material 2. [file 12864_2025_11381_MOESM2_ESM.zip › Supplementary file (Original gels and blots)/Supplementary Figure 1/PRNP off4.tif]

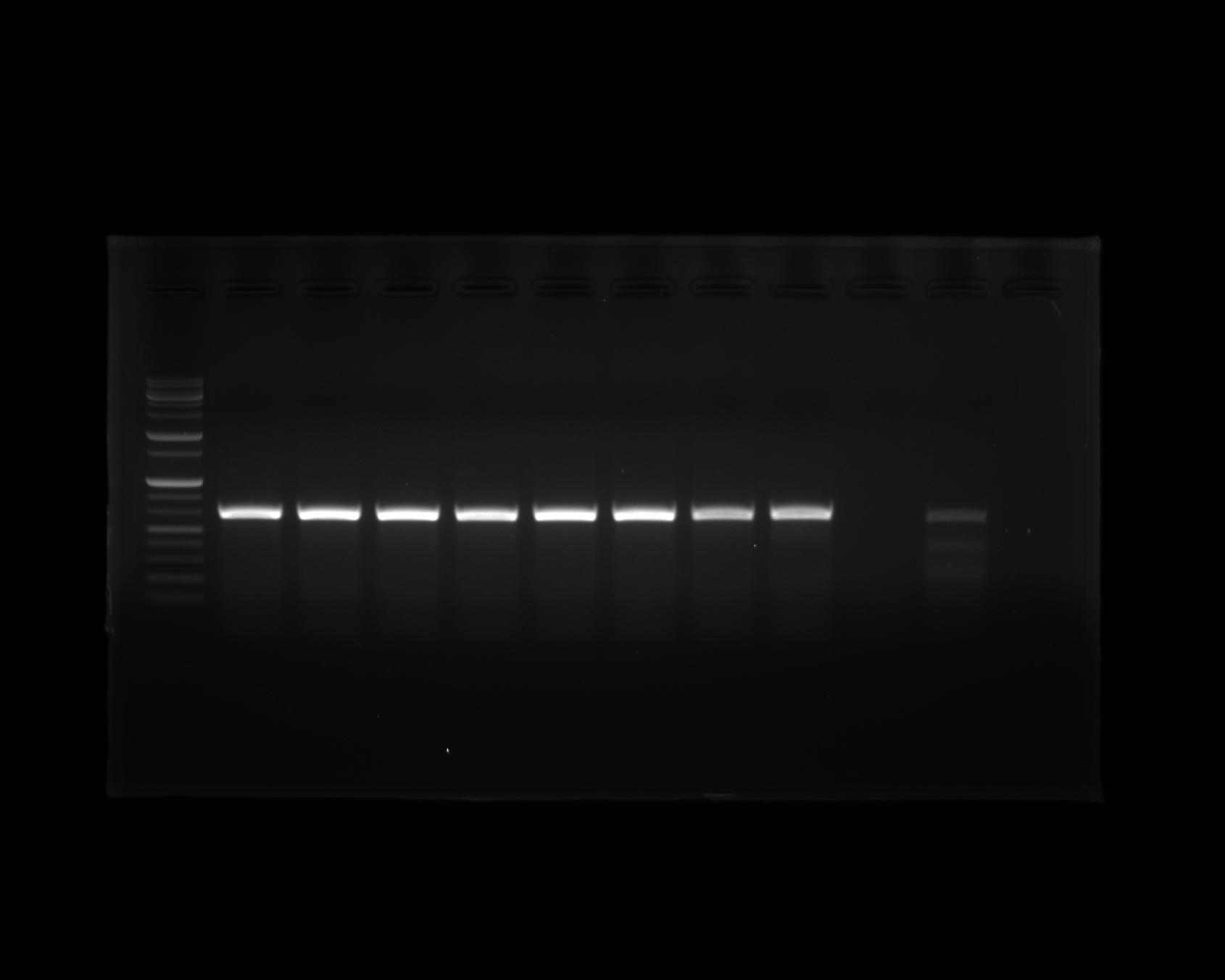

Supplement: Supplementary file 2 — Supplementary Material 2. [file 12864_2025_11381_MOESM2_ESM.zip › Supplementary file (Original gels and blots)/Supplementary Figure 1/PRNP off5.tif]

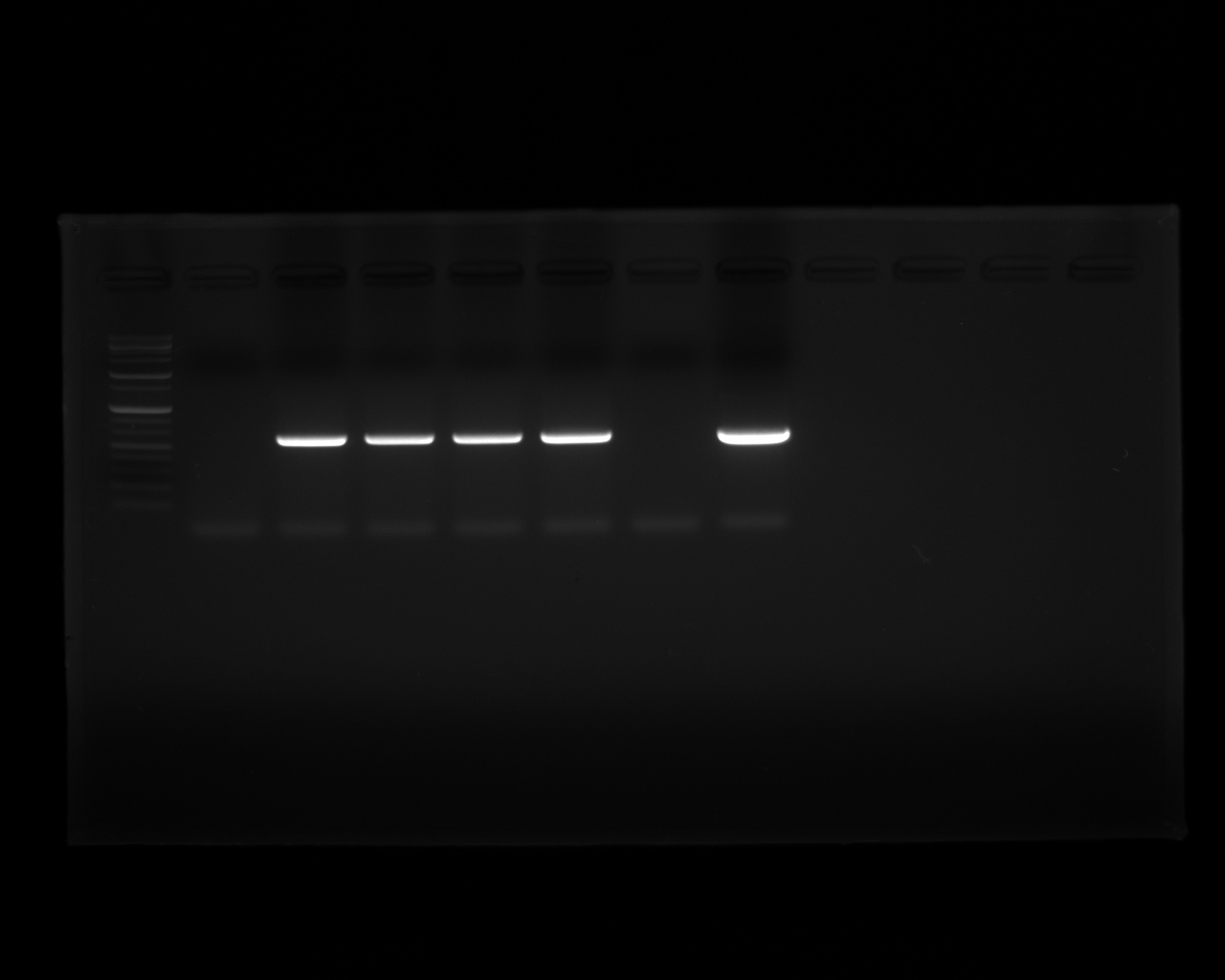

Supplement: Supplementary file 2 — Supplementary Material 2. [file 12864_2025_11381_MOESM2_ESM.zip › Supplementary file (Original gels and blots)/Supplementary Figure 2/Supplementary Figure 2A.jpg]

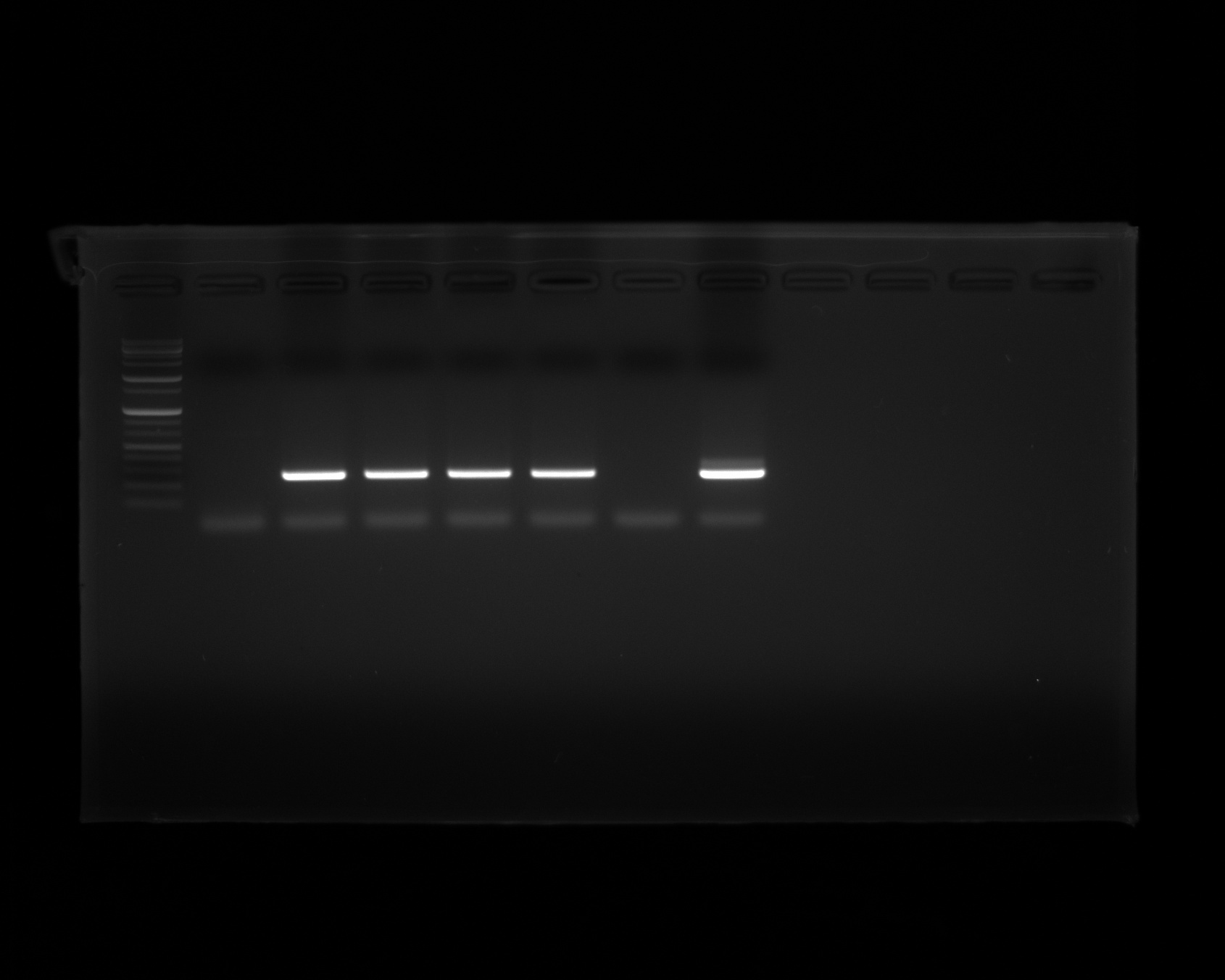

Supplement: Supplementary file 2 — Supplementary Material 2. [file 12864_2025_11381_MOESM2_ESM.zip › Supplementary file (Original gels and blots)/Supplementary Figure 2/Supplementary Figure 2B_a.jpg]

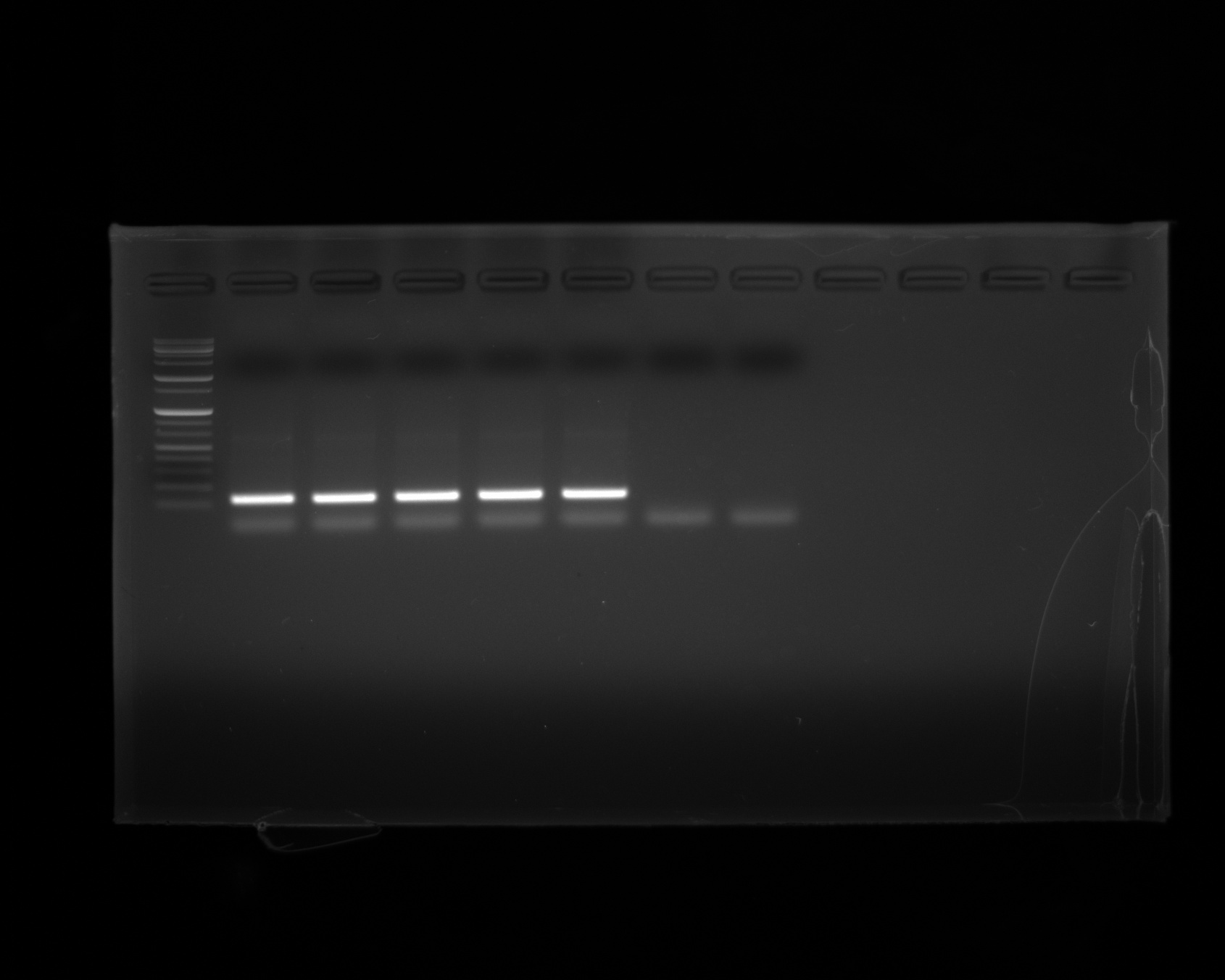

Supplement: Supplementary file 2 — Supplementary Material 2. [file 12864_2025_11381_MOESM2_ESM.zip › Supplementary file (Original gels and blots)/Supplementary Figure 2/Supplementary Figure 2B_b (from marker to 6th well).jpg]

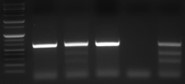

Supplement: Supplementary file 2 — Supplementary Material 2. [file 12864_2025_11381_MOESM2_ESM.zip › Supplementary file (Original gels and blots)/Supplementary Figure 4/Supplementary Figure 4A.jpg]

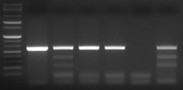

Supplement: Supplementary file 2 — Supplementary Material 2. [file 12864_2025_11381_MOESM2_ESM.zip › Supplementary file (Original gels and blots)/Supplementary Figure 4/Supplementary Figure 4B.jpg]

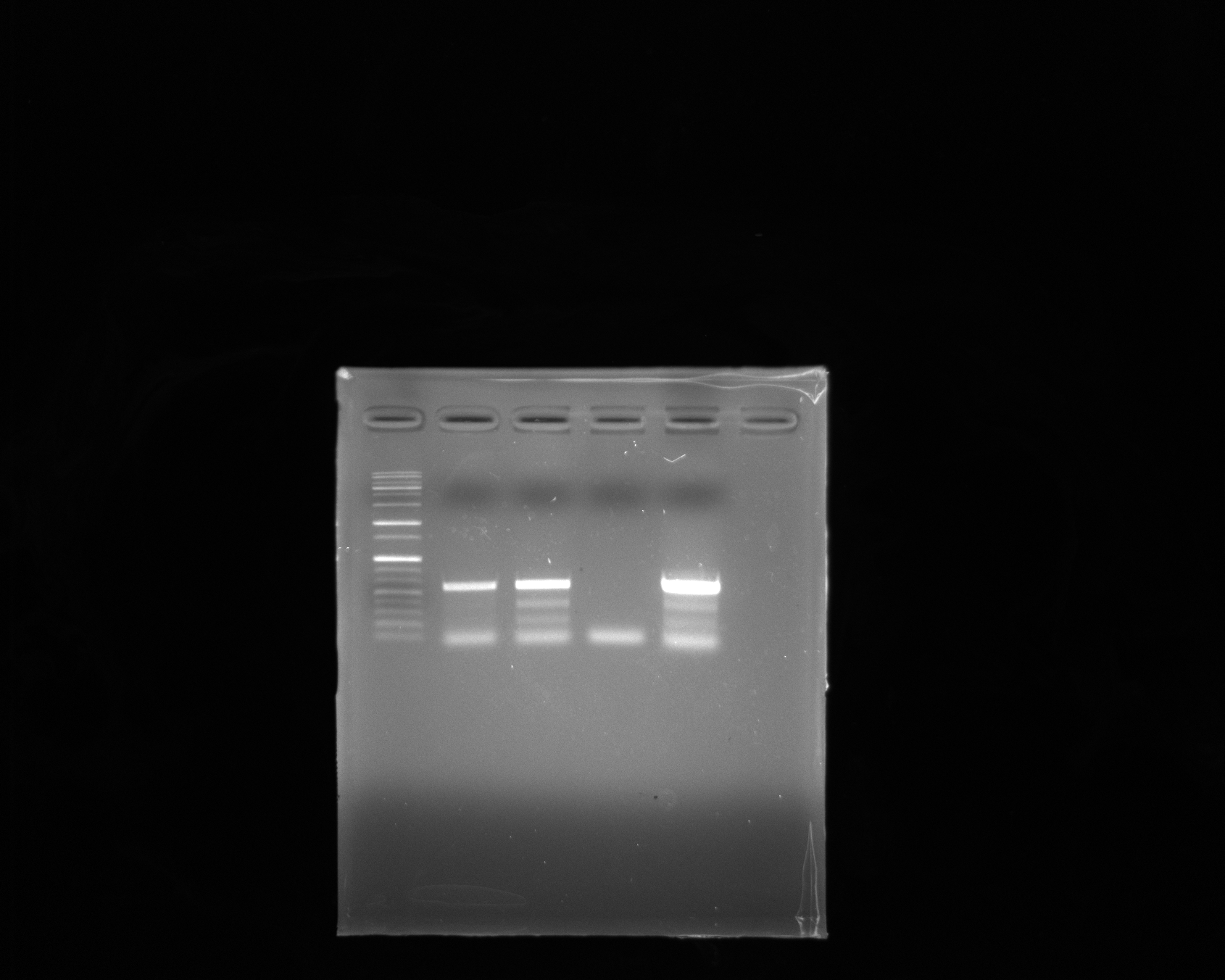

Supplement: Supplementary file 2 — Supplementary Material 2. [file 12864_2025_11381_MOESM2_ESM.zip › Supplementary file (Original gels and blots)/Supplementary Figure 4/Supplementary Figure 4C.tif]

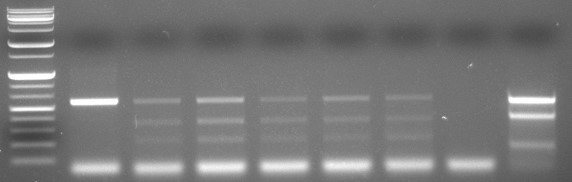

Supplement: Supplementary file 2 — Supplementary Material 2. [file 12864_2025_11381_MOESM2_ESM.zip › Supplementary file (Original gels and blots)/Supplementary Figure 4/Supplementary Figure 4D.jpg]

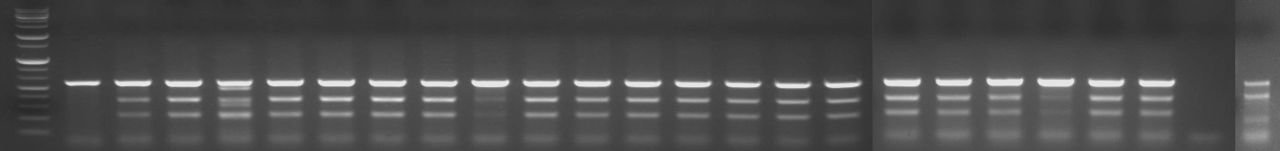

Supplement: Supplementary file 2 — Supplementary Material 2. [file 12864_2025_11381_MOESM2_ESM.zip › Supplementary file (Original gels and blots)/Supplementary Figure 4/Supplementary Figure 4E.png]
